# Role of Plant-Derived Compounds in the Molecular Pathways Related to Inflammation

**DOI:** 10.3390/ijms24054666

**Published:** 2023-02-28

**Authors:** Agata J. Olędzka, Monika E. Czerwińska

**Affiliations:** 1 Department of Biochemistry and Pharmacogenomics, Faculty of Pharmacy, Medical University of Warsaw, 1 Banacha Str., 02-097 Warsaw, Poland; 2 Centre for Preclinical Research, Medical University of Warsaw, 1B Banacha Str., 02-097 Warsaw, Poland

**Keywords:** MAPK, NF-κB, Nrf-2, phenolic compounds, phytotherapy

## Abstract

Inflammation is the primary response to infection and injury. Its beneficial effect is an immediate resolution of the pathophysiological event. However, sustained production of inflammatory mediators such as reactive oxygen species and cytokines may cause alterations in DNA integrity and lead to malignant cell transformation and cancer. More attention has recently been paid to pyroptosis, which is an inflammatory necrosis that activates inflammasomes and the secretion of cytokines. Taking into consideration that phenolic compounds are widely available in diet and medicinal plants, their role in the prevention and support of the treatment of chronic diseases is apparent. Recently, much attention has been paid to explaining the significance of isolated compounds in the molecular pathways related to inflammation. Therefore, this review aimed to screen reports concerning the molecular mode of action assigned to phenolic compounds. The most representative compounds from the classes of flavonoids, tannins, phenolic acids, and phenolic glycosides were selected for this review. Our attention was focused mainly on nuclear factor-κB (NF-κB), nuclear factor erythroid 2-related factor 2 (Nrf2), and mitogen-activated protein kinase (MAPK) signaling pathways. Literature searching was performed using Scopus, PubMed, and Medline databases. In conclusion, based on the available literature, phenolic compounds regulate NF-κB, Nrf2, and MAPK signaling, which supports their potential role in chronic inflammatory disorders, including osteoarthritis, neurodegenerative diseases, cardiovascular, and pulmonary disorders.

## 1. Introduction

Chronic inflammatory diseases or non-communicable chronic diseases have been recognized as the most significant cause of death in the world. It is worth noting that more than 50% of all deaths are due to inflammation-related diseases, such as ischemic heart disease, stroke, cancer, diabetes mellitus, chronic kidney disease, non-alcoholic fatty liver disease (NAFLD), and autoimmune and neurodegenerative conditions [1].

Inflammation is considered a protective response of the host against tissue injuries and infection. Characteristics of inflammation are vasodilation and the recruitment of immune cells and plasma proteins to the site of inflammation. The best-known definition of inflammation includes clinical symptoms such as fever (heat), dolor (pain), rubor (redness), and tumor (swelling) [2]. Inflammation is a protective immune response generated by an innate immune system after stimulation with pathogens, dead cells, or irritants [3]. The innate immune system is the first line of host defense during infection. It is crucial for the early recognition and subsequent triggering of a pro-inflammatory response to invading pathogens. On the other hand, the adaptive immune system is responsible for the elimination of specific pathogens in the late phase of infection and the generation of immunological memory [4]. From the molecular point of view, an inflammatory state means the induction of inflammasome formation as a result of the stimulation of membrane receptors. The inflammasome is defined as a cytoplasmic multiprotein complex comprising a sensor protein, inflammatory caspases, and some adapter proteins [5,6]. A normal inflammatory response is characterized by the temporally restricted upregulation of inflammatory activity that occurs during the treatment phase. The inflammatory activity is resolved after the pathological condition subsides [7]. Many environmental agents, including social, psychological, and biological factors have been linked to the promotion of a state of low-grade, non-infective, systemic, and chronic inflammation. Chronic inflammation is characterized by the activation of an immune response that is often distinct from this engaged during an acute immune response [7]. The resolution phase of inflammation includes a reduction in polymorphonuclear neutrophils (PMNs) number along with the biosynthesis of lipoxins, resolvins (E- and D-series), protectins, and maresins [8]. Lipoxins, resolvins, maresins, and protectins exert their pro-resolving actions through specific G-protein coupled receptors [9]. Human PMNs exposed to prostaglandins (PGE2, PGD2) induce RNA expression of 15-lipoxygenase (15-LOX). During inflammation, PMNs switch a phenotype. When PMNs are activated, they produce LTB4 and then switch to produce lipoxins via 15-LOX. Thus, lipoxins constitute stop signals for the limitation of further PMN recruitment [8]. The insufficient inflammatory response can lead to pathogenic infection, while excessive inflammation can cause chronic or systemic inflammatory diseases, including autoimmune disorders [3]. It is worth noting that inflammation, apart from microbial infection, is often a result of trauma, ischemia–reperfusion injury, or chemically induced injury. Therefore, this type of inflammation, which typically occurs in the absence of any microorganisms, has been named ‘sterile inflammation’. The recruitment of leukocytes and the production of pro-inflammatory agents is similar to microbially induced inflammation [10]. For this reason, the circulating, internal, sterile inflammatory signals might be responsible for chronic or even acute disorders, including atherosclerosis, cardiovascular disease (myocardial infarction, stroke), as well as neurotoxicity in Alzheimer’s disease.

It is worth noting that some signals may play a dual role, e.g., toll-like receptors (TLRs) signaling has contrasting functions on the carcinogenesis pathways. Firstly, it promotes carcinogenesis through pro-inflammatory, anti-apoptotic, proliferative, and pro-fibrogenic signals within the tumor microenvironment or tumor cells. Low and chronic stimulation of TLR2/TLR4 leads to tumor-promoting effects by enhancing inflammatory and anti-apoptotic signals. Secondly, TLRs signaling induces a sensitive and effective tumor immunosurveillance by activating specific immune cells, which have anti-tumorigenic activity. The key transcription factor NF-κB plays the central role in both these effects [11]. Currently, non-communicable chronic diseases include conditions associated with cardiovascular diseases, diabetes mellitus, chronic respiratory diseases, neurodegenerative diseases, and cancers. The background for them includes genetic and physiological conditions, as well as environmental factors. A high-quality diet rich in phenolic antioxidant compounds is related to a lower risk of non-communicable chronic diseases. Therefore, phenolic compounds are still being explored due to their therapeutical potential [12].

Plant secondary metabolites are crucial for plant interactions with the environment as well as helpful in its functionality and growth. Apart from the ecological aspect of their functions, they were first named “secondary”. Nevertheless, the definition of secondary metabolites is still changing. Due to their basic function in the response of plants to various environmental stressors, they have sometimes been named “specialized” metabolites, while primary metabolites are named “central” [13]. Phenolic compounds seem to be a wide group of secondary/specialized metabolites, and they are known for their antioxidative properties, which are mainly believed to be responsible for the reduction of inflammation. The emergency role of plant-derived products, including their antioxidative significance, should be firstly considered in the prevention of low-grade chronic inflammation. In this review, we focused on the effect of isolated plant-derived compounds on the molecular pathways, the expression of which determines the development of inflammatory response. Taking into consideration that phenolic compounds are the most abundant compounds present in medicinal and dietary plants, we particularly revised the reports concerning flavonoids, tannins, phenolic acids as well as phenolic glycosides. Literature searching was performed using Scopus, PubMed, and Medline databases without any date limitation. The terms defining the names of selected compounds and NF-κB, Nrf2, and MAPK were used to search in vitro and in vivo studies. The most relevant reports associated with chronic inflammation were included in this review. Despite many reviews paying attention to the molecular pathways themselves, we briefly described the major pathways considered in the studies of the anti-inflammatory activity of plant-derived products.

## 2. Molecular Pathways of Inflammation

The innate immune system is based on the barriers and different types of cells recognizing pathogens. Physical and chemical defense mechanisms include the epidermis, ciliated respiratory epithelium, vascular endothelium, and mucosal surfaces. The cellular components of innate immunity are represented by antigen-presenting dendritic cells, macrophages, and granulocytes capable of phagocytosis, natural killer (NK) cells, and *γδ* T lymphocytes [4].

The acute inflammatory response is typically initiated by infection via an interaction between pattern recognition receptors (PRRs), which are expressed on innate immune cells, and evolutionarily conserved structures on pathogens. These specific pathogenic molecular motifs are named pathogen-associated molecular patterns (PAMPs) [7]. They include specific molecular motifs derived from microbes such as lipopolysaccharide (LPS), flagellin, peptidoglycans, zymosan from fungi, profilin from *Toxoplasma gondi*, as well as viruses or protozoa. Additionally, the acute inflammatory response can be activated by damage-associated molecular patterns (DAMPs), which include cell-derived products. Contrary to PAMPs, DAMPs initiate immunological signals in response to trauma, ischemia, and tissue damage, even though the pathogenic infection is absent [10,14]. However, they are released in response to physical, chemical, or metabolic noxious stimuli during cellular stress or damage [7]. The representatives of DAMPs are high-mobility group box 1 (HMGB1), S100 proteins, heat shock proteins (HSPs), *β*-amyloid, and cholesterol crystals [10,14]. They are somewhat ‘sterile’ agents [7]. Therefore, systemic chronic inflammation is triggered particularly by DAMPs, which lead to metabolic dysfunction and tissue damage. Indeed, DAMPs are responsible for persistent, non-resolving, low-grade, and age-related inflammation in contrast to PAMPs linked with short-term and high-grade inflammation, the outcome of which leads to healing, trigger removal, and tissue repair. It is worth noting that IL-6, TNF-*α*, IL-1*β*, and c-reactive protein (CRP) are mainly considered biomarkers of PAMPs-related acute inflammation, whereas ‘silent’ systemic chronic inflammation is characterized by no specific markers [7]. Even though most of the models used in the studies of plant-derived metabolites use stimulators that are typical for acute inflammation, including LPS, TNF-*α*, and IL-1*β*, the relationship with chronic inflammatory disorders was most often considered in these studies.

### 2.1. Pattern Recognition Receptors

For recognition of PAMPs, the immune cells express PRRs [15]. Both PAMPs and DAMPs are recognized by five families of PRRs such as TLRs, a retinoid acid-inducible gene I (RIG-I)-like receptors (RLRs), nucleotide-binding oligomerization domain (NOD)-like receptors (NLRs), C-type lectin-like receptors, and cytosolic DNA sensors [16]. Additionally, AIM2-like receptors (ALRs) are listed among TLRs [11].

In the structure of TLRs, glycoproteins with an extracellular or luminal ligand-binding domain containing leucine-rich repeat motifs and a cytoplasmic signaling Toll/interleukin (IL)-1 receptor homology domain can be distinguished. Ten types of receptors have been identified in the family of TLRs in humans [4,17]. Depending on the number of cysteine clusters present within the extracellular leucine-rich repeat (LRR) motif, there are TLRs as follows: vertebrate-type (V-Type) and protostome-type (P-Type) [11]. They are mainly present on the cell surface (TLR1-6) or located in intracellular compartments such as endoplasmic reticulum, endosomes, and lysosomes (TLR3, -7, -8, and -9). Depending on the subfamily, they recognize lipids (TLR1, -2, -4, -6) or nucleic acids (TLR3, -7, -8, -9). TLR1/TLR2 and TLR2/TLR6 directly bind tri- or diacetylated lipopeptides. The fragments of Gram-negative bacteria such as lipid A derived from LPS interact with TLR4, whereas lipoproteins, peptidoglycan, zymosan, and *β*-glycan from fungi are detected by TLR2. On the other hand, TLR3, TLR7/8, and TLR9 are activated by double-stranded RNA (dsRNA) produced during viral replication, single-stranded RNA (ssRNA), and CpG DNA, respectively [4]. The domains of TLRs, which are critical for the functioning of the TLR signaling, include myeloid differentiation primary-response protein 88 (MyD88), TIR (Toll/IL-1 receptor) domain-containing adaptor protein (TIRAP or Mal), TIR domain-containing adaptor inducing interferon (IFN)-*β* (TRIF, TICAM1), TRIF-related adaptor molecule (TRAM, TICAM2), B-cell adaptor for phosphoinositide-3-kinase (BCAP), and sterile *α*- and armadillo-motif-containing protein (SARM) [11]. The interaction of PAMP-TLR induces receptor oligomerization, which subsequently triggers intracellular signal transduction of antimicrobial and pro-inflammatory response [4]. Toll-like receptor signaling is based on two pathways depending on the recruitment of MyD88 or TRIF [11]. The MyD88-dependent pathway leads to the enhanced expression of pro-inflammatory cytokines. The recruitment of MyD88 is facilitated by adaptor molecules such as Mal/TIRAP and TRAM in all TLRs or TLR1, -2, -4, and -6, respectively. The additional association of adaptor molecules is required to facilitate contact with MyD88 [11,18]. The formation of functional TLR4 and TLR9 signaling complexes starts when the lipid-binding domain of TIRAP binds to phosphatidylinositol 4,5-bisphosphate at the plasma membrane and phosphatidylinositol 3-phosphate on endosomes [19]. More and more proteins playing the role of co-receptors with TLRs are recognized. Glycophosphatidylinositol-anchored proteins such as CD14 induces immunoreceptor tyrosine-based activation motif (ITAM)-mediated Syk (spleen tyrosine kinase) and phospholipase C*γ*2-dependent endocytosis to promote TLR4 internalization into endosomes for activation of TRIF-dependent signaling as a result of LPS recognition [20]. Furthermore, proteins from the scavenger receptor family such as CD36 are co-receptors for oxidized low-density lipoprotein (LDL) and *β*-amyloid. It is worth noting that in liver cells, CD36 is involved in fatty acid metabolism, whereas on platelets, it is related to platelet activation. In myocardial tissue, CD36 mediates the uptake of long-chain fatty acids. The synthesis and translocation of CD36 from the endosome to the cell membrane are affected by short-term stimulation of insulin, hyperglycemia, and hyperlipidemia, while long-term stimulation induces protein synthesis. CD36 expression in macrophages is significantly increased in human carotid atherosclerotic tissue, particularly in advanced atherosclerosis, which is a progressive chronic inflammation of the arterial wall, manifested by the accumulation of foam cells, retention of macrophages in plaques, and thrombosis [21].

In general, ligands induce the oligomerization of TLR4/TLR6 heterodimers through Src kinases [18]. On the other hand, TLR3- and TLR4-TRIF-dependent pathways trigger the enhanced expression of IFN type-1 genes [11]. Therefore, TRIF signaling contributes to the promotion of inflammatory mediators and activation of immunological responses to microbes, controlling both bacterial and viral infections. In addition, the TRIF pathway plays both protective and pathologic roles in several chronic inflammatory disease conditions and wound-repair processes [22].

However, apart from TLR3 and TLR9, RLRs and NLRs can induce type I IFN (IFN-*α* and IFN-*β*) production in response to RNA, DNA, or viral infections [4]. Another family of innate immune receptors is the group of NLRs, which also recognize PAMPs. However, their structures and cellular location differ from TLRs [23]. Toll-like receptors are membrane receptors that are sensitive to extracellular microbial infections, whereas NLRs are cytosolic complexes that detect microbial products reaching the cytosol [24]. A cooperative role of TLRs and NLRs has been recognized during infection by *Escherichia coli* [25]. However, when TLR signaling fails, the activity of NLRs is intensified [26]. The family of NLRs includes NLRA, NLRB, NLRC, NLRP, and NLRX. The main members of the NLRs are NOD1 (NLRC1) and NOD2 (NLRC2) from the NLRC family [27]. NOD1 protein consists of a caspase activation and recruitment domain (CARD), a nucleotide-binding oligomerization domain (NOD), and multiple LRRs. On the other hand, the NOD2 protein possesses an additional N-terminal CARD domain [26,28]. Both proteins exist in a monomeric state in the cytosol, where they are regulated by auto-inhibition [26]. NOD1 is expressed by different cells, including epithelial cells, stromal cells, and endothelial cells, whereas NOD2 is rather limited to certain cell types such as hematopoietic cells, neutrophils, monocytes, dendritic cells, macrophages, B cells, and T cells [27,29,30]. After ligand recognition, conformational changes promote their activation. They both interact with bacterial molecules derived from the synthesis and degradation of peptidoglycan, including diaminopimelic acid (*γ*-D-glutamyl-meso-diaminopimelic acid) and muramyl dipeptide [4]. Peptidoglycan motifs are present both in gram-negative and certain gram-positive bacteria [26]. Activation of NOD proteins induces oligomerization and recruitment of downstream signaling molecules and transcriptional upregulation of inflammatory genes. Other NLR proteins are involved in the activation of caspases [4]. Peptide transporters such as SLC15A3 and SLC15A4 contribute to the transport of muramyl dipeptide across membranes of phagosomes, whereas NOD1 and NOD2 are localized [24]. Mutations at the NOD2 locus are a common risk factor in inflammatory bowel disease [24]. Caruso et al. revised the role of NOD1 in the enteroinvasive infection caused by *E. coli*, *Pseudomonas aeruginosa*, *Campylobacter jejuni*, Clostridium difficile, as well as *Legionella pneumophila*. On the other hand, the activity of NOD2 was recognized during *Listeria monocytogenes*, *Salmonella typhimurium*, *Shigella flexneri*, and *Mycobacterium tuberculosis* [26].

Changing NOD1 and NOD2 conformation leads to their self-oligomerization. Receptor-interacting serine (Ser)/threonine (Thr)-protein kinase 2 (RIPK2) is recruited through homotypic CARD–CARD interactions. Next, TAK1 Ser-Thr kinase, which is a prerequisite for activation of a multi-subunit IκB kinase (IKK) complex and mitogen-activated protein kinases (MAPK) pathway, is recruited and activated via the RIPK2 Ser-Thr kinase. The post-translation modification of proteins with ubiquitin affects many steps in the NF-κB pathway. The crucial IKK-mediated phosphorylation of the NF-κB inhibitor IκBα leads to its polyubiquitination. The proteasome allows NF-κB to translocate to the nucleus and influence the expression of target genes [26]. This molecular step is most often targeted by plant-derived compounds.

The family of NLRP proteins forms inflammasomes in response to microbial pathogens, UV light, mitochondrial reactive oxygen species (ROS) production, crystalline particles, and potassium efflux. NLRC4 is known for its sense of bacterial flagellin [27]. Activated NLRC4 and NLRP members recruit apoptosis-associated speck-like proteins (ASCs) and pro-caspase-1 to form the basis of the inflammasome. Activated caspase-1 cleaves pro-IL-1*β* and pro-IL-18 into their active forms [27,31]. Taking into consideration that the innate immune system plays a role in ischemia/reperfusion injuries in a wide range of diseases such as cardiovascular disorders, myocardial infarction, and transplantations, targeting inflammasome by plant-derived products may reduce the development of the biochemical events associated with these disorders.

### 2.2. Nuclear Factor-κB

Nuclear factor-κB (NF-κB) represents a family of inducible transcription factors. It is considered a regulator of innate and adaptive immune functions and serves as a pivotal mediator of inflammatory response. The family of NF-κB is composed of five members characterized by similar structures, including proteins p50 (NF-κB1), p52 (NF-κB2), p65 (RelA), RelB, and c-Rel. The inhibitory proteins such as IκB family members sequestrate NF-κB in the cytoplasm [16]. Major signaling pathways, such as the canonical and noncanonical named alternative, are distinguished based on signaling mechanism [32]. The canonical NF-κB pathway responds to diverse stimuli, including ligands of various cytokine receptors, PRRs, and TNF receptor (TNFR) superfamily members, as well as T-cell receptor (TCR) and B-cell receptors [16]. The background for canonical NF-κB activation is the inducible degradation of IκBα, triggered through its site-specific phosphorylation by the IKK complex [33]. This IKK complex is composed of two catalytic subunits, IKKα and IKKβ, and a regulatory subunit, IKKγ. The stimulus agents for the NF-κB activation are cytokines, growth factors, mitogens, microbial, and stress agents [33,34]. The activation of IKK first leads to the phosphorylation of N-terminal serines in IκBα. This triggers the ubiquitin-dependent degradation of IκBα. As a consequence, NF-κB members, mainly p50/RelA, are rapidly translocated to the nucleus [16]. The inhibition of phosphorylation of IκBα as well as p65 is the main target for phytochemicals, which aims to decrease the development of inflammation.

On the other hand, a non-canonical NF-κB pathway activates the p52/RelB NF-κB complex using a mechanism that relies on the inducible processing of p100 instead of the degradation of IκBα [32]. It responds to a specific group of stimuli, including ligands of a subset of TNFR superfamily members such as lymphotoxin *β* receptor (LT*β*R), B-cell activating factor receptor (BAFFR), CD40, and receptor activator of NF-κB (RANK) [32]. The processing of p100 involves degradation of its C-terminal IκB-like structure, resulting in the generation of p52 and nuclear translocation of the non-canonical NF-κB complex p52/RelB heterodimer [32].

Nuclear factor κB induces the expression of various pro-inflammatory genes in innate immune cells, including those encoding cytokines and chemokines, and also participates in activation, differentiation, and effector functions of inflammatory T cells, as well as inflammasome regulation [16]. The overexpression of pro-inflammatory cytokines (IL-1*β*, TNF-*α*, IL-6), as well as anti-apoptotic genes, are characteristics of the cancer microenvironment [11]. On the other hand, pro-apoptotic pathways mediated by c-Jun kinase are restricted [35].

Deregulation of NF-κB pathways is characteristic of chronic inflammation. Therefore, the pro-inflammatory function of NF-κB members is a therapeutic strategy in the treatment of inflammatory diseases with plant-derived products. The points of the NF-κB pathway are often targets for testing the anti-inflammatory significance of natural compounds or preparations rich in these constituents.

In particular, the pro-inflammatory function of NF-κB was considered in macrophages. However, apart from macrophages, other innate immune cells such as dendritic cells (DCs) and PMNs play significant roles in innate immunity and inflammation. After activation of NF-κB, inflammatory mediators may promote the differentiation of inflammatory T cells [16].

The receptor for LPS is mainly TLR4. Extracellular proteins, such as LPS-binding protein, CD14, and G_i_ protein participate in transferring LPS to a signaling complex composed of myeloid differentiation protein-2 (MD2) and MyD88 or mediate LPS effects [36]. The MyD88-dependent TLR pathway, and in particular, LPS through TLR4 signaling, is crucial for M1 macrophage polarization and expression of cytokines [16]. Under different conditions, activated macrophages are differentiated into phenotypically different states, including the classically activated (M1) and the alternatively activated (M2) macrophages. Macrophages M1 are induced for the production of pro-inflammatory cytokines, such as IL-1, IL-6, IL-12, IL-23, TNF-*α*, and chemokines, nitric oxide (NO), and reactive oxygen intermediates involved in various inflammatory processes [37]. The M1 macrophages also promote the differentiation of further mediating inflammation T cells such as Th1 and Th17 cells. Nuclear factor κB is a key transcription factor of M1 macrophages and is required for the induction of a large number of inflammatory genes, including those encoding TNF-*α*, IL-1*β*, IL-6, IL-12, p40, and cyclooxygenase-2 [16]. In contrast, M2 macrophages produce anti-inflammatory cytokines, such as IL-10 and IL-13, and are important for the resolution of inflammation and mediating wound-healing [16].

According to the available literature, a wide range of studies have focused on the reactivity of plant-derived products with the NF-κB system.

### 2.3. Transcription Factor Nrf2

Nuclear factor erythroid 2-related factor 2 (Nrf2) is a transcription factor that is engaged in the regulation of the cellular defense against toxic and oxidative stressors. Its main role is the protection of proteins and DNA from ROS and electrophile-derived damage. Nrf2 is expressed in all cell types. For this reason, inhibitors of Nrf2-KEAP1 interaction might be therapeutic targets for stabilizing Nrf2 in neurodegeneration, inflammation, and cancer [38].

It belongs to the subfamily of basic leucine zipper (bZIP) transcription factors, named Cap’n’Collar, along with nuclear factor erythroid-derived 2 (NFE2) and, related to it, Nrf1 and Nrf3. The Nrf2 protein is composed of seven conserved Nrf2-ECH homology (Neh) domains (Neh1 to Neh7) [39]. The bZIP proteins in Neh1 are necessary for sensing antioxidant response elements (ARE) and further activation of gene transcription. On the other hand, the E3 ligase adapter Kelch-like-ECH-associated protein 1 (Keap1) binds to the Neh2 domain containing motifs commonly known as ETGE and DLG [38]. The E3 ligase complex is formed by Cullin3 and RBX1 proteins (CUL3/RBX1). The Keap 1 mediates Nrf2 ubiquitination and degradation, or strictly presents Nrf2 for ubiquitination, which assures rather low basal protein concentration in physiological conditions out of stress [40]. The Neh3-5 domains are responsible for transactivation at the transcription level. On the other hand, the Neh7 domain interacts with the DNA-binding domain of the retinoic receptor (RXRα) [38,40]. The activation of Nrf2 can be also mediated by MAPK, such as p38, extracellular signal-regulated kinase (ERK), or c-Jun NH_2_-terminal kinase (JNK) [41].

The Keap1 protein is one of the BTB-Kelch family members known as Kelch-like 1–42 (KLHL1–42) or Kelch and BTB domain-containing 1–14 (KBTBD1–14). The Keap1 (syn. KLHL19) is composed of three domains such as N-terminal BTB (a broad complex, tram track, bric-a-brac), 3-box motif, the central intervening region (IVR domain), and the C-terminal Kelch domain [40]. The oxidative stress limits the functions of Keap1. It also takes place in the case of the presence of electrophilic xenobiotics. The sensitivity to electrophilic attack or covalent modification is due to the presence of 27 cysteine residues in the Keap1 protein, among which the most important for interactions seems to be Cys-151 in the BTB region [38,40]. The Nrf2 is accumulated in the nucleus for the activation of critical stress-response genes [38]. In the nucleus, Nrf2 forms a heterodimer with small musculoaponeurotic fibrosarcoma (Maf) proteins, which is a protooncogenic transcription factor. This dimer binds to the cis-acting ARE located in the promoter region of target genes. As a consequence, the expression of genes including phase II such as uridine 5′-diphospho-glucuronosyltransferase, glutathione transferase, NAD(P)H quinone oxidoreductase 1 (NQO1), and antioxidant enzymes such as heme oxygenase 1 (HO-1) and *γ*-glutamylcysteine synthetase are induced [41,42,43]. In the studies of phenolic compounds, the expressions of Nrf2, HO-1, and NQO1 are particularly considered as a targeting point.

Activation of Nrf2, which is equal to the inhibition of Keap1, can be a pharmacological target in certain diseases linked with oxidative stress and inflammation, such as cancer, as well as metabolic, vascular, and neurodegenerative diseases. Homeostasis through Nrf2 activators provides a beneficial therapeutic effect as well, as it is in the background of the models of most chronic disorders. The mechanism of Nrf2 activators is based on the prevention of its degradation by Keap1-dependent mechanisms. Firstly, closed-in Nrf2 complexes can be protected against ubiquitination. Secondly, another mechanism is based on the interaction of the CUL3/RBX1 complex needed for the Nrf2 ubiquitination. Thirdly, the glycogen synthase kinase 3 (GSK-3), which belongs to Ser/Thr protein kinases, and the E3 ligase adapter *β*-TrCP are responsible for the proteasomal degradation of Nrf2. Active GSK-3 phosphorylates the Neh6 domain of Nrf2 when receptor signaling is absent. Finally, several regulatory sequences, such as the xenobiotic response element, antioxidant response element, 12-*O*-tetradecanoylphorbol-13-acetate response element, and NF-κB binding site, are present in the NFE2L2 gene promoter [40]. Synthetic triterpenoids derived from oleanolic acid are characterized by the strong reactivity of Michael acceptor [40,44]. Oleanolic acid itself, as a representative of plant-derived triterpenoids, caused the increase in the mRNA expression of Nrf2, its target genes, as well as HO-1, which decreased the amounts of ROS and inflammation in wild mice [45]. Synthetic drugs, such as bardoxolone methyl, have been already included in clinical trials for the treatment of chronic kidney disease and type II diabetes mellitus, or NAFLD [40].

Among natural products, a few inducers such as quercetin, genistein, curcumin, resveratrol, and andrographolide were found to induce Nrf2 activity [40]. In particular, chronic inflammation within the gastrointestinal tract is considered a risk factor for cancer development. The consumption of a diet rich in Brassicaceae (Cruciferae) plants offers more resistance to cancer development through the activation of ARE. In particular, the breakdown products of glucosinolates are believed to play a significant health-promoting role in the prevention of cancer. Glucosinolates, such as glucoraphanin, glucoerucin, and sinigrin are the secondary metabolites of Brassicaceae. The product of their hydrolysis (thioglucosidic bond) catalyzed by myrosinase, a thioglucohydrolase (E.C. 3.2.1.147), is the bioactive isothiocyanate. Glucoraphanin is converted to sulforaphane, which is found in broccoli and red cabbage [41]. It was summarized that sulforaphane exerted beneficial effects in neurodegenerative disorders and reduced the size of infarction [40]. The methanolic aqueous extract of broccoli sprout, containing glucobrassicin, gluconasturtiin, as well as sinapic acid derivatives and flavonoids, was shown to affect the secretion of cytokines such as TNF-*α*, IL-6, and IL-10 in LPS-stimulated human peripheral blood mononuclear cells [46]. The metabolism of drugs is correlated with the transcription of proteins through Nrf2. Some studies suggest that anti-carcinogenic and anti-inflammatory properties of isothiocyanates are related to an activation of the redox-sensitive transcription Nrf2 that controls the expression of antioxidant and phase II enzymes [41].

On the other hand, Nrf2 is overexpressed in cancer cells and is linked with resistance to therapy. Cancer cells resist chemo- and radiotherapy due to Nrf2. Therefore, searching for inhibitors of Nrf2 in consideration of the sensitization of tumor cells may lead to therapy success [40].

### 2.4. Mitogen-Activated Protein Kinases

Mitogen-activated protein kinases are settled in the protein cascade, the main function of which is the regulation of gene expression, differentiation, motility, cell proliferation, and apoptosis [47,48]. Their functionality is based on the conversion of extracellular signals to a cellular response. The most common MAPK include isoforms of p38 (*α*, *β*, *γ*, and *δ*), extracellular signal-regulated kinases 1/2 (ERK1/2), c-Jun amino (N)-terminal kinases 1/2/3 (JNK1/2/3), and ERK5, while an additional group of atypical kinases is composed of ERK3/4, ERK7, and Nemo-like kinase (NLK) [48]. These kinases contain the protein Ser/Thr kinase domain, which is typical for all kinases of this family, whereas other domains, such as transactivation domains (TAD), nuclear localization sequences (NLS), C34, which is a region conserved in ERK3 and ERK4, or AHQr, which is a domain that is rich in Ala, His, and Glu, differentiate both conventional and atypical kinases. Extracellular signal-regulated kinases such as ERK1/2, ERK5, and ERK7/8 contain TEY kinase domain with Thr in positions 202 (ERK1/MAPK3), 185 (ERK2/MAPK1), 219 (ERK5/MAPK7), as well as Tyr in positions 204 (ERK1/MAPK3), 187 (ERK2/MAPK1), and 221 (ERK5/MAPK7), whereas TGY and TPY are characteristics of p38 and JNK1/2/3, respectively [47,48]. The conventional MAPKs include three evolutionarily conserved kinases: MAPK, MAPK kinase (MAPKK), and MAPKK kinase (MAPKKK). Each MAPK is activated by phosphorylation of a tripeptide motif, Thr-X-Tyr, which is located in the activation loop named T-loop. An external stimulator initiates the phosphorylation of MAPKKK, which is also considered a member of the RAF family, via interaction with a GTP-binding protein of the Ras/Rho family. Next, the cascade of phosphorylation affects MAPKK as well as Tyr and Thr residues in the conserved regions of MAPK, such as MEK1 and MEK2, which in turn activate ERK1 and ERK2 [47,49]. Phosphorylation of cytoplasmic and nuclear substrates such as transcription factors and regulatory molecules transmit signals to induce and regulate genes controlling cell proliferation or oncogenic transformation [48]. The stimulating factors, e.g., for ERK1/2, are peptide growth factors, ligands for heterotrimeric G protein-coupled receptors, cytokines, hormones, oxidative or osmotic stress, endoplasmic reticulum stress, and microtubule disorganization, as well as insulin [50]. The engagement of ERK1/2 in the cell cycle progression is possible through the stabilization of c-Fos and association with c-Jun. In this manner, the c-Jun/c-Fos dimer promotes the expression of cyclin D1, which permits G1/S transition. Furthermore, ERK1/2 mediates cell motility and the migration of tumor cells as well as fibroblasts and keratinocytes by phosphorylating actin-binding proteins implicated in cytoskeletal remodeling. This might be the reason for the progression of tumor invasion and metastasis [51].

Additional factors stimulating MAPK pathways include JNK proteins, hypoxia, UV radiation, toxins, and drugs, as well as metabolic changes associated with obesity and hyperlipidemia. Like ERK1/2, JNK proteins are also engaged in the control of apoptosis, cell proliferation, and migration [49]. Kinases such as ERK1/2 are important regulators of glucose and lipid metabolism in the liver and adipocytes, including enhanced adipocyte lipolysis by activated ERK1/2. Indeed, insulin-induced cytokine production in macrophages through ERK1/2 and IKKβ activation is able to develop insulin resistance in hepatocytes. The kinases are responsible for blocking insulin activity by inhibitory Ser phosphorylation of the insulin receptor substrate. In general, the inhibition of NF-κB and p38/ERK1/2 MAPK pathways improves insulin sensitivity [49]. However, physiological concentrations of glucose activate insulin-induced ERK1/2 activation in pancreatic *β*-cells to induce insulin gene expression. Taking into consideration that the infiltration of immune cell, macrophage-derived metabolic inflammation, and the interaction of adipocytes and macrophages are crucial factors in obesity, the conventional MAPKs are essential regulators of adipose tissue inflammation. TNF-*α* is known for its MAPK activation, leading to downstream transcriptional programs that promote pro-inflammatory gene expression. In response to JNK signaling, adipocytes of obese patients secrete HMGB1, which belongs to mediators of inflammation. In animals fed with a high-fat diet (HFD), the deletion of JNK led to the reduction hepatic production of glucose. The decreased M1 macrophage polarization in adipose tissue was observed in JNK1/2-knockout mice [52]. Inhibition of other kinases, such as p38, is able to lower blood glucose by reducing apoptosis and improving function of *β*-cells. In contrast, phosphatases inactivate kinases such as ERK1/2 through dephosphorylation, which can inhibit inflammatory gene expression. Moreover, the increased protein levels of MAPK phosphatase-3 (MKP-3) in the hypothalamus is associated with the reduction of ERK1/2 phosphorylation in the hypothalamus and leads to an increase in body weight. On the other hand, the data concerning the role of ERK1/2 or JNK1/2 in the promoting or suppressing the obesity or central metabolic role are still ambiguous, depending on animal models [49].

Deregulation of MAPK pathways has been pointed out as a possible pathogenic factor of disorders such as Alzheimer’s disease, Parkinson’s disease, diabetes mellitus, obesity, NAFLD, and cancers [49]. The role of phenolic compounds targeting MAPK pathways in neuronal and autoimmune disorders has been already revised [53,54].

**Figure 1:**
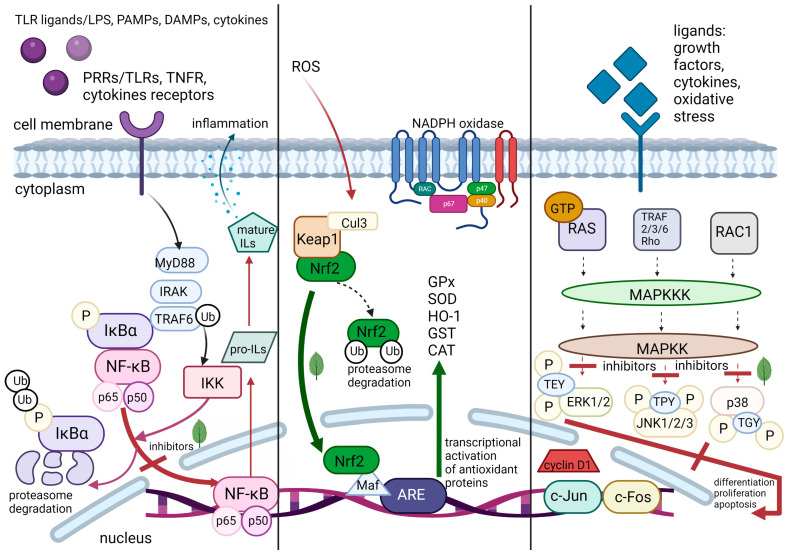
Molecular pathways engaged in inflammation. Red arrows with crossed lines—possible places of inhibition by plant-derived products; green arrows—possible places of activation by plant-derived products. Created with BioRender.com.

The major molecular pathways considered in this review as targets for plant-derived compounds are briefly presented in Figure 1.

## 3. Flavonoids

### 3.1. Flavones

**Figure 2:**
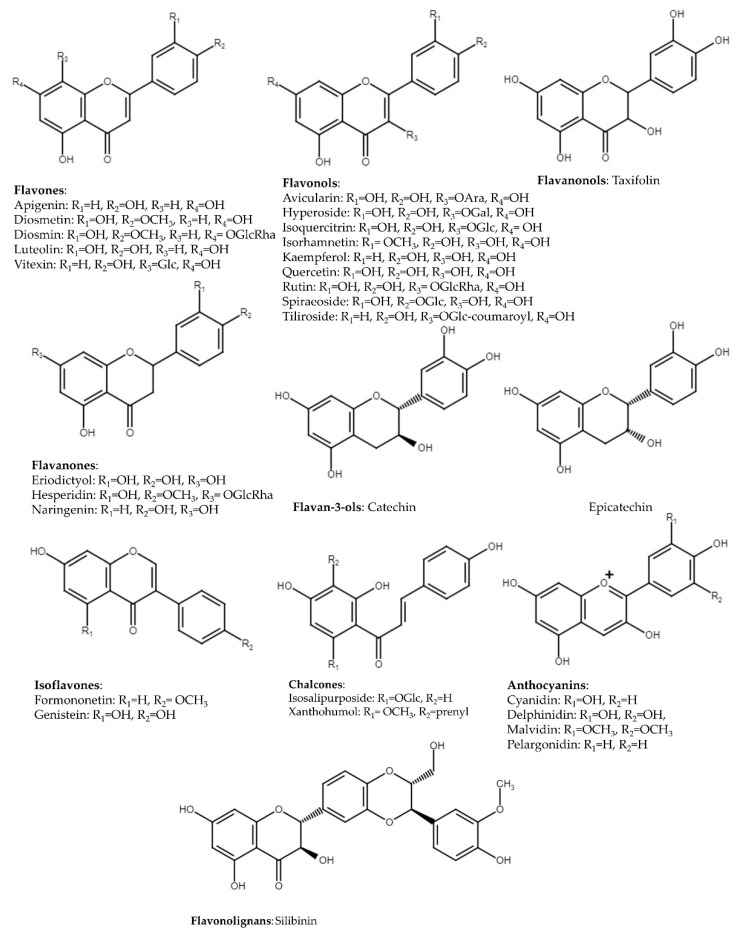
The structures of compounds listed in Table 1. Ara—arabinose; Gal—galactose; Glc—glucose; Rha—rhamnose.

Flavonoids are a particularly widely distributed class of compounds in the plant kingdom. Flavones possess a ketone in position 4 of the C ring and are characterized by the presence of a double bond (C-2/C-3) in the flavonoids skeleton and the lack of substitution at C-3. Most flavones of vegetables and fruits have a hydroxyl group in position 5 of the A ring. On the other hand, hydroxylation in other positions as in position 7 of the A ring or 3′ and 4′ of the B ring is taxonomically dependent (Figure 2).

Flavones are pigments of white and cream flowers, and they play a protective role against UVB due to their capability of UV absorption in the range from 280 to 315 nm. Plants from Lamiaceae, Asteraceae, and Apiaceae, as well as fruits and fruit juices such as bergamot, grapefruit, mandarin orange, orange, and citron juices, are the most abundant sources of flavones. In addition, flavones are present in celery, parsley, red peppers, chamomile, and mint [55,56]. High concentrations of flavones can be detected in fresh foods. As was described previously, flavones are often present in plants and fruit juices as glycosides. However, free aglycones were detected in teas and dry herbs as well [56]. We selected apigenin, diosmetin, and luteolin, as well as glycosides such as diosmin (diosmetin 7-*O*-rutinoside) and vitexin (apigenin-8-*C*-glucoside), as representatives of flavones. In general, their inhibitory activities of NF-κB and MAPK signaling as well as induction of Nrf2 were established in different in vitro and in vivo models.

Apigenin at the concentration of 5 µM significantly inhibited TGF-*β*1-induced phosphorylation of p50, p38, and JNK in contrast to ERK in nasal fibroblasts from patients suffering from chronic rhinosinusitis ex vivo. It is worth noting that the expression and translocation of p50 as a subunit of NF-κB were comparably inhibited by apigenin as p38 inhibitor (SB203580; 10 μM) and JNK inhibitor (SP600125; 5 μM). In the wound-scratch assay, apigenin inhibited TGF-*β*1-induced fibroblast migration. The suppression of TGF-*β*1-induced myofibroblasts and extracellular matrix production (fibronectin, collagen type I) in nasal inferior turbinate tissues ex vivo by apigenin (10 μM) was due to the inhibition of MAPK and NF-κB signaling. Taking into consideration that excessive tissue remodeling and the activation of nasal fibroblasts can lead to the pathogenesis of disease by inducing tissue fibrosis associated with chronic rhinosinusitis, tissue remodeling in long-term disorders might be a therapeutic target for plant-derived products [57].

It is believed that there is a crosstalk between ROS and immune responses, which can be regulated by the balance between Nrf2 and NF-κB. The antioxidative activity of flavones such as apigenin occurs through the upregulation and activation of Nrf2 signaling. Apigenin in concentrations from 1 to 40 µM increased mRNA levels of Nrf2, HO-1, and NQO1 in human melanocytes (PIG3V) and murine BV2 microglial cells [58,59]. In addition, oxidative stress caused by H_2_O_2_ was suppressed by apigenin, which elevated the levels of enzymes belonging to the antioxidant defense system (ADS) such as sodium dismutase (SOD), catalase (CAT), and glutathione peroxidase (GSH-Px) [58]. In addition, apigenin in concentrations of 10–40 µM significantly inhibited the production of TNF-*α*, IL-6, and IL-1*β* by murine BV2 microglial cells. On the other hand, LPS induces nuclear translocation of p65 NF-κB, and treatment with apigenin attenuated this process and reduced the amount of p65 NF-κB in the nucleus [59]. These findings were confirmed in vivo in the LPS-induced lung injury in mice both for apigenin and its *C*-glucoside, vitexin, as well as for diosmetin [60,61,62]. Furthermore, vitexin at doses of 15, 30, and 60 mg/kg inactivated MAPK and NF-κB signaling pathways through the reduction of the protein expression of p38, ERK, and JNK, as well as the p65 subunit of NF-κB in *Staphylococcus aureus*-induced mastitis in mice [63]. In the same model, luteolin at doses of 25, 50, and 100 mg/kg reduced protein expression of IκB in addition to p65. It is worth noting that luteolin also caused a decrease in the mRNA expression of TLR2 and TLR4 in mammary gland tissues [64]. Diosmin is known for its vein-protective properties. It strengthens the tension of venous walls by increasing the contractility of smooth muscles under the influence of calcium ions [65]. Both diosmin and diosmetin significantly inhibited elastase and collagenase activities as well as cytokine production in LPS-induced human fibroblasts of BJs cell line [66]. Diosmin was first isolated in 1925 from *Scrophularia nodosa* L. (Scrophulariaceae) and has been widely used since 1969 due to its phlebotonic and vascular protective properties in the treatment of venous leg ulcers and hemorrhoids. Most of the data support its use for chronic venous insufficiency, as well as acute and chronic hemorrhoid disease [67,68,69,70]. In the commercial product, diosmin (90%) is combined in a micronized form with hesperidin (10%), referred to as micronized purified flavonoid fraction (MPFF). It is usually administered for 3 months or less [69]. Doses of both 500 mg twice a day and 1000 mg MPFF were effective in the reduction of leg pain in chronic venous disease [67]. Taking into consideration the bioavailability of diosmin, it is hydrolyzed by the gut microbiome in the intestine to diosmetin, which is further absorbed and eliminated in the urine as diosmetin-3-*O*-glucuronide [71]. Therefore, it seems that the role of aglycone forms should be particularly considered in vitro and in vivo. On the other hand, the digestion with gut microbiota enzymes may provide data on the potential metabolites formed in the gastrointestinal tract from the plant-derived compounds [72].

The summary of the studies concerning the effect of flavonoids and other classes of phenolic compounds in molecular pathways is presented in Table 1 and Table 2, respectively.

**Table 1:** Summary of studies concerning the role of flavonoids in molecular pathways; ↓ inhibition; ↑ activation.

Compounds	Model	Mode of Action	Reference
Flavones			
Apigenin	TGF-*β*1-induced nasal fibroblasts	↓ MAPK, NF-κB	[57]
	human melanocytes (PIG3V)	↑ Nrf2	[58]
	murine microglial cell line (BV2)	↑ Nrf2, ↓ NF-κB, TNF-*α*, IL-1*β*, IL-6	[59]
	LPS-induced acute lung injury in mice	↓ NF-κB, TNF-*α*, IL-1*β*, IL-6, COX-2	[60]
	coculture of adipocytes (3T3-L1) and macrophages (RAW 264.7)	↓ NF-κB, MAPK	[73]
Diosmetin	*S. pneumoniae*-induced meningitis in rats	↓ NF-κB, TNF-*α*, IL-1*β*, IL-6	[74]
	LPS-induced acute lung injury in mice	↑ Nrf2, ↓ TNF-*α*, IL-1*β*, IL-6	[61]
Diosmin	alloxan-induced nephropathy in rats	↓ NF-κB, TNF-*α*, IL-1*β*, IL-6	[75]
	doxorubicin-induced hepatotoxicity in rats	↓ NF-κB, MAPK, TNF-*α*, IL-6, IL-1*β*	[76]
Luteolin	monolayer of colon adenocarcinoma cell line (Caco-2)	↑ Nrf2, ↓ NF-κB, MAPK	[77]
	*S. aureus*-induced mastitis in mice	↓ NF-κB, TNF-*α*, IL-1*β*, IL-6, MMP-2, MMP-9	[64]
	LPS-induced ulcerative colitis in rats	↓ NF-κB, IL-17, IL-23	[78]
Vitexin	rat insulinoma cell line (INS-1)	↑ Nrf2, ↓ NF-κB	[79]
	LPS-induced acute lung injury in mice	↑ Nrf2	[62]
	S. *aureus*-induced mastitis in mice	↓ NF-κB, MAPK, TNF-*α*, IL-1*β*, IL-6	[63]
Flavonols			
Avicularin	IL-1*β*-induced rat and human chondrocytes	↓ MAPK, iNOS, COX-2, MMP3, MMP-13, TRAF-6	[80]
	IL-1*β*-induced murine chondrocytes	↑ Nrf2, ↓ NF-κB, COX-2	[81]
Hyperoside	TNF-*α*-induced intervertebral disc degeneration in human nucleus pulposus cells	↑ Nrf2, ↓ NF-κB, IL-6, iNOS, COX-2, TNF-*α*, IL-1*β*, MMP-3, MMP-13	[82]
	human lung carcinoma cell line (A549)	↓MAPK	[83]
	OVA *-induced asthma model in mice	↑ Nrf2, ↓ NF-κB, IL-4, IL-5, IL-13	[84]
Isoquercitrin	human liver cancer cell line (HepG2)	↓ MAPK	[85]
	acute myocardial infraction model in rats	↓ NF-κB, TNF-*α*, IL-6, NO	[86]
	middle cerebral artery occlusion in rats	↑ Nrf2, ↓ NF-κB	[87]
Isorhamnetin	bronchial epithelial cell line (BEAS-2B)	↓ MAPK, NF-κB, IL-1*β*, IL-8, IL-6	[88]
	LPS-induced chronic obstructive pulmonary disease in mice	↑ Nrf2, ↓ IL-6	[89]
	LPS-induced lung injury in mice	↓ NF-κB, IL-6, IL-1*β*, TNF-*α*	[90]
Kaempferol	LPS- & ATP-induced cardiac fibroblasts	↓ NF-κB, TNF-*α*, IL-1*β*, IL-6, IL-18	[91]
	I/R **-induced lung injury in rats	↓ NF-κB, TNF-*α*, IL-6, HMGB1	[92]
	cadmium-chloride-induced nephropathy in rats	↑ Nrf2, ↓ NF-κB, ROS, IL-6, TNF-*α*	[93]
	murine microglial cell line (BV2) spinal cord injury model in rats	↓ MAPK, IL-1*β*, IL-18, Nox4,	[94]
Quercetin	IL-1*β*-induced nucleus pulposus cells	↑ Nrf2, ↓ NF-κB, IL-6, IL-8, MMP-3, MMP13	[95]
	leptin-induced human umbilical vein endothelial cell line (HUVEC)	↓ NF-κB, TNF-*α*	[96]
	chronic prostatitis/chronic pelvic pain syndrome model in rats	↓MAPK, NF-κB, IL-1*β*, IL-2, IL-6, IL-17A, TNF-*α*, MCP-1	[97]
	cardiomyopathic rats induced by doxorubicin	↑ Nrf2	[98]
	CAD *** patients	↓ NF-κB, TNF-*α*, IL-1*β*	[99]
Rutin	LPS-induced murine macrophages (RAW 264.7)	↓ NF-κB	[100]
	H_2_O_2_-induced oxidative stress in HUVEC	↑ Nrf2, ↓ NF-κB	[101]
	HMGB1-induced HUVEC	↓ NF-κB, VCAM-1, TNF-*α*, IL-6	[102]
	LPS-induced acute lung injury model in mice	↓MAPK, TNF-*α*, IL-6, IL-1*β*	[103]
	chronic colitis model in mice	↓ NF-κB, TNF-*α*, IL-1*β*, IL-6	[104]
Spiraeoside	human cardiomyocytes (AC16)	↑ Nrf2	[105]
Tiliroside	murine microglial cell line (BV2)	↑ Nrf2, ↓ NF-κB, IL-6, TNF-*α*, IL-1*β*	[106]
	bone-marrow-derived macrophage (BMM)	↓ NF-κB, MAPK, ROS	[107]
Flavanones			
Eriodictyol	human osteoarthritis chondrocytes	↑ Nrf2, ↓ NF-κB, IL-6, TNF-*α*, MMP-3, MMP-13	[108]
	LPS-induced microglial cell line (BV2)	↑ Nrf2, ↓ MAPK, COX-2	[109]
Hesperidin	chronic obstructive pulmonary disease model in mice	↓ NF-κB, IL-6, IL-8	[110]
	As_2_O_3_-induced cardiotoxicity in mice	↑ Nrf2, ↓ IL-6, ROS, TNF-*α*	[111]
	human neuroblastoma cell line (SH-SY5Y)	↓ MAPK	[112]
Naringenin	cardiomyocytes (H9C2)	↑ Nrf2, ↓ IL-6, IL-1*β*, TNF-*α*, MPO	[113]
	LPS-induced normal human bronchial epithelial cell line (NHBE)	↓ NF-κB, IL-6, TNF-*α*, MPO, NO	[114]
Flavanonols			
Taxifolin	acute alcohol-induced liver injury in mice	↓ NF-κB, IL-1*β*, IL-6, TNF-*α*	[115]
	benzo[a]pyrene-induced lung injury in mice	↑ Nrf2, ↓ NF-κB, TNF-*α*, COX-2	[116]
	Iron-overload-induced hepatocellular injury in rats	↓ MAPK, IL-1*β*, IL-6, TNF-*α*	[117]
Flavan-3-ols			
Catechin	*Porphyromonas gingivalis*-stimulated human monocytes (THP-1)	↓ NF-κB, MAPK, pro-IL-1*β*, TLR2, TLR4, NLRP3	[118]
	LPS-induced murine macrophages (RAW 264.7)	↓ NF-κB, MAPK, TNF-*α*, COX-2, iNOS	[119]
Epicatechin	high-glucose-induced human monocytes (THP-1)	↓ NF-κB, TNF-*α*	[120]
	LPS-induced acute lung injury model in mice	↓ MAPK, TNF-*α*, IL-6	[121]
	hemoglobin toxicity in primary astrocytes from wild type and Nrf2−/− mice	↑ Nrf2, ↓ ROS	[122]
Isoflavones			
Formononetin	human neuroblastoma cell line (SH-SY5Y)	↑ Nrf2, ↓ MAPK, ROS	[123]
	gastric ulcer model in rats	↓ NF-κB, IL-6, IL-1*β*, TNF-*α*, MPO	[124]
Genistein	homocysteine-induced inflammatory injury in endothelial cell (ECV-304)	↓ NF-κB, IL-6	[125]
	liver granuloma and fibrosis model in mice	↓ NF-κB, IL-1*β*, TNF-*α*, IL-4, IL-10	[126]
	diabetic nephropathy model in rats	↓ NF-κB, MAPK	[127]
Chalcones			
Isosalipurposide	oxidative injury in human hepatocarcinoma cell line (HepG2)	↑ Nrf2	[128]
Xanthohumol	LPS-induced acute lung injury in mice	↑ Nrf2, ↓ NF-κB, IL-6, IL-1*β*, TNF-*α*	[129]
	LPS-induced depressive-like symptoms in mice	↑ Nrf2, ↓ NF-κB	[130]
Flavonolignans			
Silibinin	LPS-induced murine macrophages (RAW 264.7)	↓ MAPK	[131]
	human colorectal cell lines (SW480, HT-29, LoVo)	↓ NF-κB, COX-2, MMP-9	[132]
	neuroblastoma cell line (SH-SY5Y)	↑ Nrf2	[133]
Anthocyanins			
Cyanidin	palmitate-induced lipotoxicity in human colon adenoracinoma cell line (Caco-2)	↑ Nrf2, ↓ NF-κB, IL-6, IL-8, COX-2	[134]
	human colon cancer cell lines (HCT116, HT29, SW620)	↑ Nrf2, ↓ NF-κB	[135]
	human nucleus pulposus cells	↑ Nrf2	[136]
	pulmonary artery hypertension model in rats	↓ MAPK, IL-6, TNF-*α*	[137]
Delphinidin	murine embryonic stem cell lines (ES-E14TG2a, ES-R1)	↓ NF-κB	[138]
	H_2_O_2_-induced human retinal pigment epithelial cell line (ARPE-19)	↑ Nrf2	[139]
Malvidin	osteoarthritis in rats, primary rat chondrocytes	↓ NF-κB, TNF-*α*, IL-1*β*, IL-6, MMP-3, MMP-9, MMP-13	[140]
	sepsis-related acute liver injury model in mice	↑ Nrf2, TNF-α, IL-1*β*, IL-6	[141]
Pelargonidin	LPS-induced murine macrophage cell line (J774)	↓ NF-κB, NO	[142]
	middle cerebral artery occlusion (MCAO) in rats	↑ Nrf2, ↓ TNF-α, IL-6	[143]

* OVA—ovalbumin; ** I/R—ischemia-reperfusion; *** CAD—coronary artery disease.

### 3.2. Flavonols

Like flavones, flavonols are very diverse in methylation and hydroxylation, as well as glycosylation patterns. They are probably the most common and the largest subgroup of flavonoids in fruits and vegetables. Flavonols are particularly distributed in onions, kale, lettuce, tomatoes, apples, grapes, and different berries, as well as in tea and red wine. The most studied flavonols seem to be quercetin, kaempferol, myricetin, and isorhamnetin. Compared with flavones, flavonols have a hydroxyl group in position 3 of the C ring. In this position, the sugar moieties are linked, as it is in the case of avicularin (quercetin 3-*O*-*α*-L-arabinoside), hyperoside (quercetin 3-*O*-*β*-D-galactoside), and isoquercitrin (quercetin 3-*O*-*β*-D-glucoside). In particular, quercetin is present in many medicinal and food plants [55]. It is the most abundantly present in onions, capers, apples, tomatoes, grapes, Brassica vegetables, and shallots. In traditional medicine, onion extracts rich in quercetin have been used externally to relieve allergic symptoms after itching [144]. It is worth noting that allergic airway diseases are chronic disorders characterized by inflammatory cell infiltration, airway hyper-responsivity, and airway inflammation. In particular, the increased number of eosinophils as well as the production of Th2 cytokines such as IL-4, IL-5, and IL-13 were observed in patients with asthma. The regulating role of Nrf2 in the Th1/Th2 balance in the lung was indicated [84]. The activity of quercetin in allergic asthma, allergic rhinitis, and atopic dermatitis and in particular, the effect on immunological aspects of asthma, such as cytokine levels, recruitment of leukocytes, and regulation of T1/T2 balance, was previously reviewed by Jafarinia et al. (2020). Based on that review, it was concluded that quercetin could regulate Th1/Th2 stability and decrease the antigen-specific IgE antibody released by B cells [145]. Quercetin in the glycosidic form of hyperoside reduced the level of cytokines typical for asthma, IgE, as well as phosphorylation of p65 NF-κB and IκBα. On the other hand, hyperoside increased Nrf2 and HO-1 in ovalbumin (OVA)-induced asthma in BALB/c mice [84]. A similar anti-inflammatory effect of isorhamnetin (10–40 µM), including MAPK and NF-κB signaling pathways, was established in human bronchial epithelial BEAS-2B cells [88].

Moreover, flavonoids such as quercetin can be considered senolytic agents. Taking into consideration the development of musculoskeletal diseases in modern society, the role of quercetin in intervertebral disc degeneration (IDD), which is the main reason for low back pain, has been reported. The gelatinous internal nucleus pulposus (NP) cells are one of the types of tissue cells in the intervertebral disc. Quercetin in concentrations from 10 to 20 µM inhibited the protein expression of p65 NF-κB as well as the nucleus level of p65 in NP cells. In this study, the IκBα level was higher in the cells treated at 20 µM but lower than in the non-treated control [95]. On the other hand, quercetin reduced the expression of the IκBα and further decreased the transcriptional activity of NF-κB and IL-1*β* in stable coronary artery disease in 85 patients. It is worth noting that IL-1*β* is related to TNF receptor-associated factor 6 (TRAF6) action on IKKα, which separates the complex of p50/p65 NF-κB with IκBα, leading to the translocation of the p65 subunit into the nucleus, where the final transcription of the genes of various inflammatory molecules takes place [99]. Moreover, the nucleus level of Nrf2, as well as the level of HO-1 in the NP cells, were increasing when the NP cells were treated with quercetin [95]. Similarly, quercetin glycoside such as hyperoside at the concentrations of 10, 20, and 50 µM reduced inflammation by decreasing IL-1*β*, IL-6, inducible nitric oxide synthase (iNOS), COX-2, NF-κB protein expression, as well as extracellular matrix protein (MMP-3, MMP-13) degradation but increasing Nrf2, HO-1, and NQO1 in NP cells [82]. On the other hand, tiliroside prevented bone loss in ovariectomy (OVX) in mice and inhibited osteoclast differentiation and bone resorption stimulated by receptor activator of NF-κB ligand (RANKL) in vitro. Tiliroside in the concentration range of 10–60 µM significantly reduced ROS production and phosphorylation of p65 NF-κB as well as phosphorylation of MAPK proteins in RANKL-induced bone marrow-derived macrophages (BMM) [107].

In addition to musculoskeletal disorders, the next important problem of modern society is obesity. In obesity conditions, the concentration of leptin is often high in plasma. By the binding of leptin to its receptor, signal transduction pathways of inflammation are activated. The release of excess inflammatory factors affects endothelial dysfunction that could lead to cardiovascular disease and serious consequences of metabolic syndrome. The beneficial effects of quercetin (125 µM) in the down-regulation of the leptin receptor expression, reduction of ERK1/2 phosphorylation, and inhibition of NF-κB activation in leptin-induced human umbilical vein endothelial cells (HUVEC) were established [96]. Furthermore, quercetin significantly reversed doxorubicin-induced biochemical, histopathological, hemodynamic, physical, and physiological parameters in rodent cardiomyopathy [98]. In particular, quercetin at a dose of 25 mg/kg significantly activated Nrf2 expression. Apart from endothelial inflammation in atherosclerosis, chronic inflammation might be, on the other hand, the first step of cancer pathogenesis, depending on the target tissue. Chronic prostatitis or chronic pelvic pain syndrome are the most commonly found chronic urinary tract diseases. Quercetin at the doses of 50, 100, and 200 mg/kg decreased the tissue concentration of the main inflammatory cytokines such as IL-1*β*, IL-2, IL-6, IL-17A, MCP1, and TNF-*α* in rats. The phosphorylation of p65 NF-κB, p38 MAPK, ERK, and JNK were significantly lowered, depending on the dose [97].

Apart from quercetin itself, many studies have researched the activity of quercetin glycosides both in vitro and in vivo. Taking into consideration the potential hydrolysis of glycosides in the gut, their pharmacological effect should be concluded based on in vivo studies. Since cardiovascular diseases constitute the first reason for mortality in modern society, the regular intake of flavonoids may particularly provide beneficial health effects. Ischemia–reperfusion (I/R) in both heart and lung tissues can be an inducive factor for oxidative stress, inflammation, and cell damage. Kaempferol at the concentration of 12.5 and 25 µg/mL reduced LPS and ATP-induced myocardial fibroblast inflammation by inhibiting NF-κB signaling pathway activity by regulating p65 and IκBα [91]. Similarly, it improved lung injury induced by I/R in rats via decreased phosphorylation of p65 NF-κB expression in lungs and reduced HMGB1 [92]. Home-box protein 1, which belongs to the aforementioned class of DAMPs, is mainly located in the nucleus, and when the cell is damaged, it is secreted to the extracellular environment to participate in the regulation of inflammatory response through NF-κB activation [92]. For this reason, HMGB1 might be one of the factors responsible for chronic inflammation.

It is believed that long-term inflammation, when the inflammatory process is activated even if there is no external cause of injury or it is not correctly ended after infection, may lead to DNA damage and cancer. Abnormal tissue reactions or pathological conditions like in obesity are a risk factor for cancer development. Patients suffering from chronic inflammatory bowel diseases, such as ulcerative colitis and Crohn’s disease, have an increased risk of colon cancer [146]. The anti-tumor role of flavonoids through inhibition of the MAPK pathway was considered. Different signalings such as NF-κB, MAPKs, PI3K/Akt/GSK3*β*, Ras/Raf/MEK/ERK, and p38/MK2 were particularly studied to explain the mechanism of colitis or neuroinflammation. Based on the study with dextran sulfate sodium (DSS)-induced colitis in mice model, rutin was indicated as a nutraceutical agent, which may prevent intestinal inflammation. Rutin in a dose of 25 mg/kg decreased the activation of p38, ERK, and JNK, as well as diminished p-NF-κB and NF-κB protein expression. It is worth noting that rutin augmented intestinal structural and functional homeostasis in colon tissues through mucin 3 protein expression [104]. Contrary to traditional apoptosis, pyroptosis is defined as a kind of inflammatory necrosis, which activates inflammasomes, including NLRP3, and subsequently the secretion of cytokines such as IL-1*β* and IL-18. Inflammasomes play a role in the host defense system, and NLRP3 is one of the inflammasomes engaged in neurodegenerative diseases. Kaempferol at doses of 25, 50, and 100 mg/kg regulated ROS-dependent MAPK–NF-κB and NLRP3-mediated pyroptosis signaling pathway via the decrease in the expression levels of NADPH oxidase (Nox) 4, IL-1*β*, p-p38, p-JNK, and NLRP3 in spinal cord injury in rats and/or in BV2 cells [94].

However, in most of the studies, the effect on signaling pathways such as MAPK and PKC was evaluated only in cancer cells and was not related to the effect in the normal cell lines. Hyperoside in the concentration range of 1 to 5 µM inhibited the phosphorylation of p38 MAPK in A549 lung cancer cells. Therefore, it can be engaged in the invasion and metastasis of many tumors [83]. Isoquercitrin (200–400 µM) inhibited liver cancer cells (HepG2) through a decrease in the phosphorylation of all main MAPK, such as p38, ERK1/2, and JNK [85]. Nevertheless, the mechanism of the inhibition of tumor growth observed in vivo requires further elucidation.

### 3.3. Flavanones

Flavanones, which are named dihydroflavones, have a double bond between positions C-2 and C-3 in a saturated C ring. Flavanones are another important class that is generally present in all citrus fruits such as oranges, lemons, and grapes. Eriodictyol, naringenin, and hesperidin are examples of flavanones [55].

First, naringenin in the concentration of 10–40 µM inhibited the mRNA expression of crucial inflammatory factors such as IL-6, TNF-*α*, NO, and NOS in normal human bronchial epithelial (NHBE) cells stimulated with LPS. The most significant effect in the inhibition of p65 NF-κB expression was observed in the case of naringenin at the concentration of 10 µM. The MAPK signal transduction via inhibition of JNK and p38 was reduced by naringenin in a concentration-dependent manner in this model [114]. In addition, the role of naringenin in ferroptosis was studied by the investigation of myocardial I/R-injured rats and H/R-induced H9C2 cells. Similar to pyroptosis, ferroptosis is a new type of cell death that is related to oxidative stress, like in diabetic MIRI and endoplasmic reticulum stress. Naringenin reduced the size of myocardial infarction as well as inflammation and lipid peroxidation in rodent cardiac tissues. It increased Nrf2 and decreased Nox1 expression in H9C2 cells [113].

The influence of eriodictyol and hesperidin on chronic inflammation pathways in osteoarthritis and neuroinflammation models was studied. Eriodictyol in the concentration range 6.25–25 µM decreased mRNA expression or secretion of inflammatory factors such as PGE2, NO, IL-6, TNF-*α*, MMP-3, and MMP-13 by IL-1*β*- stimulated chondrocytes isolated from articular cartilage samples ex vivo. The compound at the highest tested concentration in this study decreased phosphorylation of p65 NF-κB, while the expression of Nrf2 and HO-1 was increased [108]. Next, eriodictyol (50 µM) and hesperidin (20 µM) protected neuronal cells such as BV2 and SH−SY5Y from high glucose-induced oxidative stress by inhibition of phospho-JNK, phospho-ERK2, and phospho-p38, respectively [109,112]. Furthermore, hesperidin at doses of 100–300 mg/kg protected murine cardiomyocytes against arsenic trioxide (As_2_O_3_) stimulation in vivo through the regulation of the p62-Keap1-Nrf2 signaling pathway. Hesperidin increased the nuclear level of Nrf2 and p62, whereas Keap1 expression was reduced [111]. In lung tissues of C57BL/6 mice, hesperidin decreased phosphorylation of p65 NF-κB and PGC-1*α*. In addition, it relieved oxidative stress and inflammatory response via inhibition of the decrease in IL-6, IL-8, and myeloperoxidase (MPO), which provided support for its significance in chronic obstructive pulmonary disease in vivo [110].

### 3.4. Flavanonols

Taxifolin (dihydroquercetin) is a representative of flavanonols characterized by the lack of a double bond between the C-2 and C-3 positions of the flavone skeleton. Taxifolin showed a hepatoprotective effect at doses of 20, 40, and 80 mg/kg via reduction of liver aminotransferases induced by alcohol in mice. In particular, taxifolin at a dose of 80 mg/kg decreased the phosphorylation of IKKβ in acute alcohol-induced liver inflammatory injury in mice [115]. Moreover, the administration of taxifolin reversed the effects of iron overload and resulted in a significant decrease in the liver iron content (56% of the iron group), a reduction in apoptotic marker caspase-3 activity (53% of the iron group), and a significant increase in the protein level of PI3K p100*α*. Taxifolin reduced iron-induced phosphorylation of p38 MAPK and c-Fos to 75% and 69% of the iron-treated control rats, respectively. In addition, it down-regulated the levels of TNF-*α*, IL-1*β*, and IL-6 to 50%, 52%, and 49% of the control iron-treated rats, respectively [117]. In vitro taxifolin reversed the effects of chromium (VI) by inhibiting the activation of MAPKs such as p38 and JNK, as well as IκB and p65 NF-κB. It regulated the expression of apoptosis-related proteins and alleviated the adhesion of THP-1 cells to human umbilical vein endothelial cells (HUVEC) [147]. Finally, taxifolin in doses of 20 and 40 mg/kg increased nuclear Nrf2, NQO1, HO-1, and SOD in benzo[a]pyrene-induced lung injury in male Swiss Albino mice [116]. A quite similar effect was observed in cisplatin-induced nephrotoxicity in mice [148].

### 3.5. Flavan-3-ols

Flavanols, also referred to flavan-3-ols, are characterized by the presence of the hydroxyl group, which is bound to position 3 of the C ring [55]. The two chiral centers at C-2 and C-3 of the flavan-3-ols provide four isomers for each level of B-ring hydroxylation. Among them, (+)-catechin and (−)-epicatechin are widespread, whereas (−)-catechin and (+)-epicatechin are less widespread [149]. It is worth noting that (−)-epicatechin in humans is excreted partially as (+)-epicatechin, which indicates possible ring opening and racemization in the gastrointestinal tract. On the other hand, transformation can also occur during food processing [149]. Indeed, catechin and epicatechin are the most abundant flavonoids in green tea along with their derivatives such as tannins, as well as in grapes, cocoa, and many other fruits and vegetables. The anti-inflammatory activity of these compounds was tested in vitro in human or murine monocyte/macrophage cell lines such as RAW264.7 and THP-1 [118,119,120]. The major virulence factors of *Porphyromonas gingivalis* such as LPS, fimbriae, and gingipains facilitate colonization in the oral cavity. Pretreatment of THP-1 cells with catechin (40 µM) inhibited the pro-IL-1𝛽 expression induced by *P. gingivalis* through the NF-κB, MAPK, and TLR signaling pathways. It is worth noting that the expression of all TLR-related proteins such as TLR2, TLR4, MyD88, TRIF, and TRAF6 was decreased. Apart from the transcription of pro-IL-1𝛽 via NF-κB activation by TLR, the formation of inflammasome to convert pro-IL-1𝛽 to mature IL-1𝛽 is the second pathway participating in IL-1*β* secretion. It was discovered that catechin significantly reduced the expression of NLRP3 inflammasome [118]. Taking into consideration that the metabolic dysregulation, which manifests with high plasma glucose concentration, high glucose (25 mM) was used as the inducer of inflammation. In this condition, (−)-epicatechin at a concentration of 5 µM reduced the activation of NF-κB through inhibition of phospho-p65 [120].

(−)-Epicatechin crosses the brain barrier, and it was established that it promoted cytoplasmatic and nuclear Nrf2 expression in primary astrocytes from wild-type and Nrfs knockout mice. However, epicatechin (100 μM) did not alter HO-1 level in both types of astrocytes. On the other hand, the compound decreased the phosphorylation of JNK and prevented JNK translocation. The cells were induced with hemoglobin at the concentration of 10 µM. Based on this study, epicatechin was preliminarily indicated as a preventive compound in intracerebral hemorrhage [122]. It is believed that epicatechin alleviates the progression of tissue damage. The compound reduced alveolocapillary membrane permeability and lung edema in the LPS-induced acute lung injury (ALI) model in mice. It was supposed that epicatechin might inhibit the production of inflammatory mediators in the lungs of mice with ALI by blocking p38 MAPK-mediated activation of AP1 [121].

### 3.6. Isoflavones

Isoflavones are the group of flavonoids in which the B ring is linked in position 3 of the C ring [55]. The cytoprotective and anti-inflammatory activities were established for formononetin, daidzein, and genistein, which are known for their significance in supplements for menopausal women due to their estrogenic effect [150]

Formonentin is an isoflavonoid abundantly found in medicinal herbs such as *Trifolium pratense* L. and *Astragalus membranaceus* (Fisch.) Bunge (Moench). An increase in Nrf2, NQO1, and HO-1 was observed in H_2_O_2_-induced SH-SY5Y neuroblastoma cells after treatment with formononetin (50 µM) in vitro, as well as decreases in p65 NF-κB, TNF-*α*, IL-1*β*, IL-6, and MPO in tissues of gastric ulcers in Sprague–Dawley rats treated with formononetin at doses of 25 and 50 mg/kg [123,124]. On the other hand, genistein downregulated the expressions of Nox4, MAPK, p65, and p53 in diabetic nephropathy in Sprague–Dawley rats [127]. It also reduced p65 and p-IκBα along with cytokine mRNA expression levels in the liver tissue of mice infected with *Schistosoma japonicum*. Therefore, the authors concluded that a therapeutic use for genistein was found for the prevention of hepatic granuloma and fibrosis [126].

### 3.7. Chalcones

Chalcones are open-chain flavonoids, because the ‘ring C’ of the basic flavonoid skeleton is not present in their structure. This class of compounds occurs in tomatoes, pears, strawberries, bearberries, and certain wheat products. Examples of chalcones include phloridzin, phloretin, and chalconaringenin, as well as isosalipurposide [55].

Isosalipurposide in the concentration range of 3 to 100 µM increased the nuclear level of Nrf2 in HepG2 cells. The cytoprotective effects were supported by an increase in HO-1 expression as well as the GSH/GSSG ratio [128]. A similar effect on Nrf2 signaling was established for another chalcone, xanthohumol, in HepG2 and THLE-2 cells as well as in vivo in LPS (0.5 mg/kg)-induced acute lung injury in mice. Xanthohumol is the prenylflavonoid found in the hop (*Humulus lupulus* L., Cannabaceae) [129,151]. Moreover, xanthohumol was reported to prevent liver steatosis and fibrosis in diabetes mellitus by mediating Nrf2/AGE/RAGE/NF-κB signaling [152]. The anti-inflammatory and antioxidant properties of xanthohumol are particularly appreciated due to their utility for preserving and flavoring beer, which means potentially quite high consumption. Xanthohumol effectively alleviated lung injury by reduction of neutrophil infiltration, MDA, and MPO formation, as well as SOD and GSH increase. Additionally, the compound inhibited ROS generation, cytokine secretion, iNOS, and HMGB1 expressions, as well as NLRP3 inflammasome and NF-κB signaling pathway activation in vivo [129]. In general, the role of chalcones in inflammation through NF-κB signaling was previously reviewed [153]. Nevertheless, direct studies of isosalipurposide on this molecular pathway were not provided. However, it was shown that LPS activated NF-κB (p-p65) in the hippocampus of mice and xanthohumol reduced the level of p-NF-κB in comparison with the LPS group. The authors speculated that this molecular pathway may be involved in the behavioral impairment and therapeutic base for the prevention and treatment of depression with xanthohumol-rich products [130].

### 3.8. Flavonolignans

Non-alcoholic fatty liver disease includes non-alcoholic fatty liver, steatohepatitis, fibrosis, and cirrhosis, which can lead to hepatocellular carcinoma. The disorder is characterized by the accumulation of lipids in the liver, and it is commonly associated with metabolic syndromes such as diabetes and obesity [154]. The anti-inflammatory and antifibrotic effects of silymarin were clinically proven and revised by the Committee on Herbal Medicinal Products (EMA/HMPC/318925/2017) for the European Medicine Agency in their opinion on *Sylibi mariani fructus*. It is believed that milk thistle (*Sylibum marianum* (L.) Gaertner, Asteraceae) extract may prevent hepatic fibrosis through suppression of inflammation and hypoxia in the hepatic fibrogenesis [155]. Apart from fatty acids, flavonolignans are the most abundant compounds of *Sylibi mariani fructus*. The complex of flavonolignans known as silymarin is composed of flavonol derivatives such as silibinin and isosilibinin A and B, silicristin, and silidianin [155]. Silymarin at the dose of 50 mg/kg reduced the accumulation of collagen in the liver by 30–35% after oral administration for several weeks and prevented weight increase in the liver and spleen when secondary fibrosis was increased [156]. The nominal content of silymarin is in the range of 30 to 65%, and it is mostly represented by silibinin. The content of silymarin corresponds to the sum of the contents of silibinin A and silibinin B, in the range of 40–65%, with reference to total silymarin. Silibinin A and B are diastereoisomers. In the commercially available standard silibinin, an almost equal ratio of silibinin A and B is present [157]. In the referred studies, the commercial standard of silbinin was tested. For this reason, it is cited as it was in the references without distinguishing between silibinin A and B. Additionally, the crucial case concerning silibinin is the partial absorption from the gastrointestinal tract (23–47%), which is the consequence of poor hydrosolubility, the presence of other compounds, or degradation by gastric fluid [155]. Silibinin was found to inhibit the activation of NF-κB by inhibition of IκBα, p50, or p65 phosphorylation in different cell types such as HMC-1 human mast cells, RANKL-induced RAW264.7 cells, colorectal cancer cells, and LO2 cells in vitro, as well as in monocytes from preeclamptic women ex vivo [132,158,159,160,161,162]. In addition, silibinin increased HO-1 in the hippocampus and activated Nrf2 translocation from the cytoplasm to the nucleus in formaldehyde-treated mice, as well as in H_2_O_2_-induced SH-SY5Y human neuroblastoma cells [133,163]. The inhibitory effect of silibinin (50 µg/mL) on p38 MAPK phosphorylation was established, which is important in the regulation of LPS-induced macrophages [131].

### 3.9. Anthocyanins

Anthocyanins possess flavonoid-like skeletons with a cation charge at position 1 of the C ring. In plant tissues, anthocyanidins form sugar conjugates known as anthocyanins. In addition, the anthocyanins form conjugates with hydroxycinnamates and organic acids such as malic and acetic acids. Conjugation occurs most often on C-3, although it can also take place on C5 and C7 [149]. Anthocyanins are pigments responsible for the red, violet, and blue colors of flowers, fruits, and other plant parts. They occur predominantly in fruits such as cranberries, black currants, red grapes, merlot grapes, raspberries, strawberries, blueberries, bilberries, and blackberries. The color of the anthocyanin depends on the pH, methylation, or acylation at the hydroxyl groups on the A and B rings [55]. It is assumed that the change in color in these berries would be due to the mutation in structural or regulatory genes involved in anthocyanin biosynthesis. In general, anthocyanins are synthesized in the phenylpropanoid pathway. Anthocyanin biosynthetic pathway is significantly related to flavonols, proanthocyanidins, and anthocyanidins. Therefore, it is believed that anthocyanins are biogenetically related to these classes of phytochemicals. Two classes of genes are required for anthocyanin biosynthesis. Firstly, the structural genes encode the enzymes that directly participate in the formation of anthocyanins and other flavonoids. Secondly, the regulatory genes control the transcription of structural genes. The activities of enzymes, such as dihydroflavonol 4-reductase, anthocyanidin synthase, UDP glucosyl-flavonoid 3-*O*-glucosyltransferase, and methyltransferase, in the various branch pathways through dihydroquercetin and dihydromyricetin, are highly regulated. Transcriptional controls play an important role in regulating the overall activity of flavonoid biosynthesis in response to different developmental and environmental factors [164].

Cyanidin, delphinidin, malvidin, pelargonidin, and peonidin belong to the most often studied anthocyanidins [55]. They are present in the plant as glycosides of hydroxylated 2-phenylbenzopyrylium salts (flavylium salts). Cleavage by acid hydrolysis produces the corresponding free flavylium salt [130].

Currently, obesity and inflammatory bowel disease are widely occurring disorders, and high consumption of n–6 polyunsaturated, saturated acids, and glucose is observed. Free fatty acids are spectacularly elevated in the serum of these patients. This parameter correlates with increased BMI as well as with the concentration of circulating IL-6 and TNF-*α*. In the in vitro studies, palmitic acid was used as an inducer of p65 nuclear translocation in human adenocarcinoma Caco-2 epithelial cells. Cyanidin-3-*O*-glucoside in the concentration range of 10–20 µM prevented this palmitate-induced effect and activation of the NF-κB inflammatory pathway in addition to the regulation of redox homeostasis via increased Nrf2 and NQO1 mRNA expressions. The effect of these molecular activities was observed in the inhibition of COX-2, IL-6, and IL-8 mRNA expression [134]. Similarly, in human colon cancer, HCT116, HT29, and SW620 cell lines, a significant decrease in factors related to NF-κB signaling such as nuclear p65, p50, p-IKKα/β, and p-IκBα was observed when the cells were treated with cyanidin chloride at a concentration of 50 µM [135]. On the other hand, cyanidin-3-*O*-glucoside attenuated the high-glucose-induced apoptosis and induced the expression of Nrf2 and HO-1 in human nucleus pulposus cells (HNPC) [136]. The protective effect of cyanidin-3-*O*-glucoside (400 mg/kg) on hemodynamics in rats with pulmonary artery hypertension induced by monocrotaline was studied. It was established that the compound inhibited vascular remodeling through the inhibition of p38 MAPK expression [137]. The alleviation of histopathological damage of liver tissue was observed in male C57 mice treated with malvidin. Considering molecular pathways, malvidin prevented LPS-induced reduction of SOD, GSH-PX, and CAT through up-regulation of Nrf2 and decreased mRNA levels of TNF-*α*, IL-1*β*, and IL-6, as well as protein levels of NLRP3 inflammasome in the liver [141]. The potential effectiveness of malvidin in chronic diseases was taken into consideration while this compound at the doses 10 and 20 mg/kg exerted the pain-relieving effect in osteoarthritis in rats by decreasing inflammatory cytokines and metalloproteinases, as well as suppressing the nuclear translocation of p65 via an IκBα-independent manner in primary rat chondrocytes [140]. On the other hand, the effect of delphinidin on Nrf2, JNK, and NF-κB was established in vitro in H_2_O_2_-treated human retinal pigment epithelium ARPE-19 cells concerning age-related macular degeneration (AMD) and in murine embryonic stem cell lines such as ES-E14TG2a and ES-R1 [138,139]. It is worth noting that the pathogenesis of AMD is still unknown. It is supposed that oxidative-stress-induced dysfunction of retinal pigment epithelium cells leads to secondary photoreceptor loss in the early AMD stage. Therefore, this is an important reason for blindness among the elderly population worldwide, and anthocyanins show potential in the prevention of eye disorders [139].

As far as pelargonidin is concerned, among a few flavonoids, a 100 µM dose inhibited p65 nuclear translocation and NO production (by 59 ± 0.8%) in LPS-induced J774 macrophages. In this comparative study, genistein, kaempferol, quercetin, and daidzein inhibited activation of STAT-1 and NF-κB for iNOS production, whereas four compounds, isorhamnetin, naringenin, and pelargonidin, inhibited only NF-κB signaling [142]. The rat middle cerebral artery occlusion (MCAO) model was selected to evaluate the neuroprotective effect of pelargonidin (10–20 mg/kg) on cerebral I/R injury. The authors concluded that ameliorated neurological function caused by pelargonidin treatment was through Nrf2 and HO-1 overexpression in brain tissue [143].

## 4. Lignans

Reports on phenolic compounds other than flavonoids are provided in Table 2. Firstly, the class of lignans was included. The classic representative of this group of phytochemicals is nordihydroguaiaretic acid (nordihydroguaretic acid; NDGA). It is a known, naturally occurring 5-lipoxygenase inhibitor, which was isolated from creosote (*Larrea divaricate* Cav., Zygophyllaceae) [165]. It inhibited NF-κB activation in TNF-*α* and IL-1*α*-induced Jurkat E6.1 and EL4.NOB-1 cells, as well as UVB-induced keratinocytes [166,167]. On the other hand, it was shown that NDGA along with indomethacin did not affect the TNF-induced NF-κB DNA binding activity in Jurkat and HEK293 cells. It was concluded that different inhibitors of arachidonic acid metabolism might interfere at different points in TNF-induced signaling, leading to NF-κB-dependent transcription [166,168]. In another study by Won et al. (2005), it was established that NDGA inhibited LPS-induced activation of p-NF-κB-secreted alkaline phosphatase in glial cells, suggesting the role of lipoxygenase in NF-κB-mediated iNOS gene regulation [169]. Nordihydroguaiaretic acid (30 mg/kg/day) decreased the expression of NF-κB, TNF-*α*, and phosphorylation of p38 in AP mice, which supports its role in the prevention against acute pancreatitis development [170]. Taking into consideration the chronic inflammation of skin barriers, NDGA decreased protease activated receptor 2 (PAR2)-induced inflammatory responses, including IL-8 and ICAM-1 expression in keratinocytes, and recovered skin barrier and atopic dermatitis in hairless mice [171]. Despite the lack of direct antioxidant properties, NDGA was able to induce in vivo renal Nrf2 nuclear translocation, and therefore it may be involved in the protection of this compound against I–R injury in rats [172]. In IGF-1-induced SH-SY5Y neuroblastoma cells, NDGA in concentrations of 3, 30, and 60 µM reduced the phosphorylation of ERK1/2. It activated caspase and apoptosis pathways as well as inhibited IGF-1-stimulated motility and neuroblastoma tumor growth in vivo [173]. Nordihydroguaiaretic acid prevented the activation of AP-1 and NF-κB by 2,3,7,8-tetrachlorodibenzo-*p*-dioxin, which is an agonist of the aromatic hydrocarbon receptor, a potent tumor promoter, and a liver carcinogen [174]. This might support its chemopreventive role.

## 5. Tannins

### 5.1. Gallotannins and Ellagitannins

**Figure 3:**
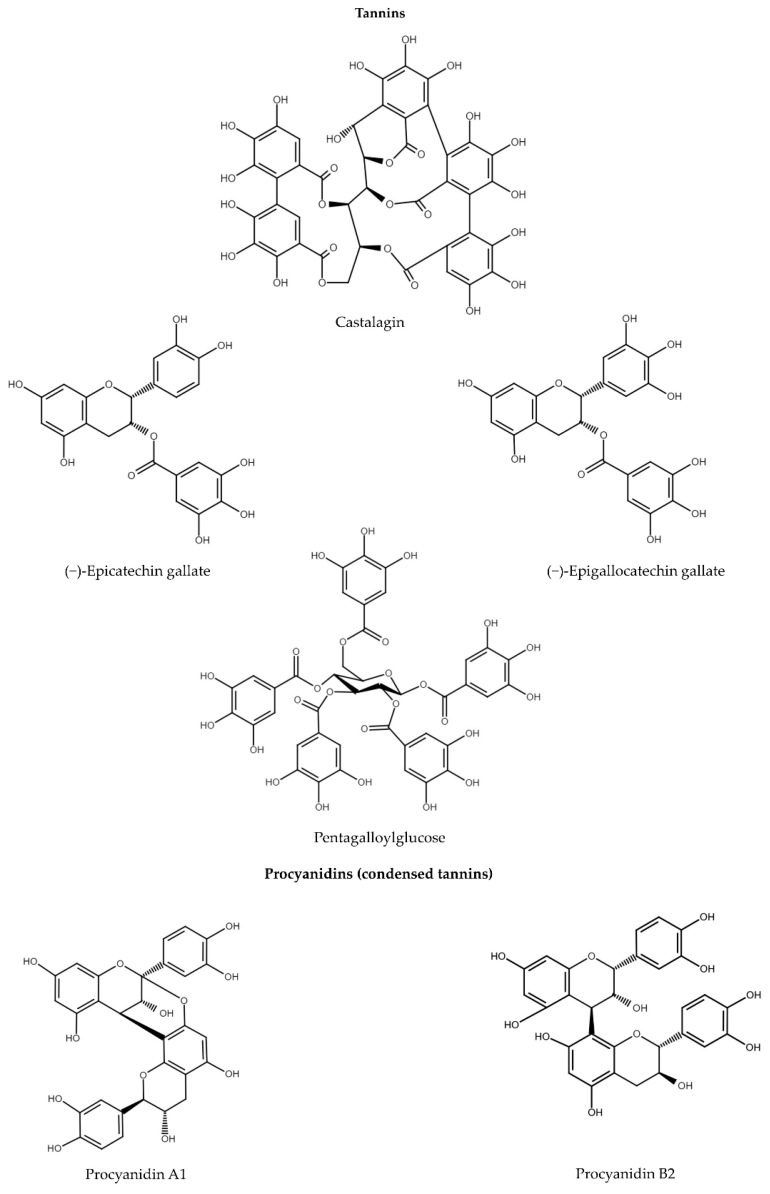
The structures of tannins listed in Table 2.

Flavan-3-ols undergo esterification with gallic acid to form gallocatechins (Figure 3). In particular, high levels of flavan-3-ols, principally (−)-epigallocatechin, (−)-epigallocatechin gallate, and (−)-epicatechin gallate are found in green tea (*Camellia sinensis* (L.) Kuntze, Theaceae). Nevertheless, during the fermentation of the tea leaves, the levels of catechins decline. Therefore, the main components in black tea are the high molecular-weight thearubigins and the less concentrated theaflavins [149]. In addition to tea polyphenols, many ellagitannins are significant players in everyday diets. Ellagitannins are compounds characterized by a high molecular weight composed of hexahydroxydiphenoyl (HHDP) moieties esterified with glucose or galloyl molecules. Polymeric structures such as punicalagin, castalagin, vescalagin, and granatin are the main representatives of ellagitannins. They are mainly characterized by the presence of a *C*-glycoside bond. Punicalin and punicalagin belong to the ellagitannins formed from HHDP linked to the glucopyranose core, whereas in castalagin-type compounds, e.g., castalagin and vescalagin, the flavogalloyl moiety is linked to the *C*-glycosidic fraction [175]. Among them, the influence on NF-κB and Nrf2 signaling pathways was particularly reported for punicalagin [176,177,178,179,180,181], whereas the number of studies concerning castaligin and vescalagin is seriously limited. This might be correlated with the metabolites of ellagic acid and ellagitannins formed by gut microbiota activity. The most commonly known metabolites brought by human gut microbiota are urolithins. They are considered the most potentially involved in protection against many diseases, such as cancer, cardiovascular diseases, neurological disorders, diabetes, and inflammatory diseases [175]. The NF-κB and Nrf2-based molecular mechanisms of the activity of urolithins A and B were provided in a few reports [182,183,184,185,186,187].

The role of hydrolyzable tannins such as castalagin and (−)-epigallocatechin-3-gallate in processes related to osteoclastogenesis was studied [188,189]. Castalagin, even at a concentration of 1 µM, significantly inhibited bone-resorbing activity. In RANKL-induced intracellular signaling during the osteoclasts differentiation from bone marrow macrophages (BMM), it blocked the phosphorylation of Akt, ERK, and p38 and, to a lesser extent, the phosphorylation of JNK and IκBα [188]. Apart from the aforementioned MAPK the phosphorylation of IKKα/β, IκBα, and p65 was inhibited by (−)-epigallocatechin-3-gallate [189]. Next, both epigallocatechin gallate and (−)-epicatechin gallate exerted positive effects in hypoxia or CoCl_2_-induced microglial cells through suppression of the NF-κB pathway and activation of Nrf-2/HO-1 [190,191]. Furthermore, through these signaling pathways, (−)-epicatechin gallate might be engaged in the prevention of atherosclerosis. (−)-Epicatechin gallate inhibited the inflammatory response, proliferation, migration, and formation of vascular smooth muscle cells (VSMC), which was induced by ox-LDL [192]. (−)-Epicatechin gallate regulated all parameters, such as MDA level and SOD expression, as well as Nrf2/HO-1 signaling, which was dysregulated upon oxidative stress in ApoE−/− mice induced by HFD or VSMC. (−)-Epicatechin gallate affected the HFD-induced inflammatory response by inhibiting the activation of the NF-κB signaling pathway in the aorta of ApoE−/− mice. It is worth noting that the safe dose of (−)-epicatechin gallate is 338 mg per day for an adult. If the daily dose is greater than 800 mg, which corresponds to 1315–1500 mg catechins per day, hepatotoxic adverse effects are possible [193]. On the other hand, epicatechin derivatives even at low doses were protective agents in acute lung or kidney injuries induced by LPS or cisplatin [194,195]. In the case of chronic kidney disorders, diabetic nephropathy is one of the most common and serious complications of diabetes, which leads to chronic kidney disease and end-stage renal disease. Hyperglycemia causes the overproduction of ROS as well as advanced glycation end products (AGE). Pentagalloylglucose (1,2,3,4,6-penta-*O*-galloyl-*β*-D-glucose) in the concentration range of 5 to 20 µM upregulated Nrf2 and HO-1 expression in AGE-induced mouse mesangial cells (MES). It was concluded that this process was related to the inhibition of JAK2/STAT3 cascade [196].

Aging as well as skin damage due to UV radiation were the targets for pentagalloylglucose. Taking into consideration the environmental factors affecting human skin, it is particularly important to prevent oxidative stress and inflammation caused by solar radiation. Plant-derived products are excellent candidates, particularly as ingredients of cosmetics and traditional preparations. As far as pentagalloylglucose is concerned, it inhibited the phosphorylation of IκBα and p65 in the NF-κB signaling pathway as well as the protein kinases of the MAPK pathway in human fibroblasts. The UVB-induced expression of COX-2 and ICAM-1 was also suppressed by the compound in a concentration-dependent manner from 1 to 10 µM [197].

### 5.2. Procyanidins

Proanthocyanidins that consist exclusively of (epi)catechin units are named procyanidins. They are the most abundant type of proanthocyanidins in plants. The oligomeric and polymeric proanthocyanidins are known as condensed tannins. Both oligomeric and polymeric proanthocyanidins have an additional chiral center at C-4. Type-B proanthocyanidins are formed from (+)-catechin and (−)-epicatechin with oxidative coupling occurring between the C-4 of the heterocycle and the C-6 or C-8 positions of the adjacent unit. Type A proanthocyanidins have an additional ether bond between C-2 and C-7. The number of units necessary for the formation of polymers is up to 50 [149]. The oligomeric procyanidins and prodelphinidins from the seeds of black grapes or the roasted seeds of cocoa (*Theobroma cacao* L., Malvaceae) occur in red wines or dark chocolate [149].

It can be noticed that RAW264.7, a monocyte/macrophage-like cell line, as well as the human monocytic leukemia THP-1 cell line, are often used in vitro for screening anti-inflammatory activity from natural compounds. These cells are commonly induced by LPS from *E. coli* O111:B4 [198,199]. Lipopolysaccharide significantly upregulates the protein expression levels of p-p65 NF-κB, p-IκBα, and p-IκBβ compared with the vehicle control groups, e.g., in HUVEC. It is worth noting that the expression of COX-2 has been also shown to be linked to MAPK signaling cascades. Indeed, COX-2 induction occurs through a JNK/c-Jun-dependent mechanism after administration of the neurotoxin. Therefore, COX-2 may play a role in the neuropathology of Parkinson’s disease. Procyanidin B2 at a concentration of 50 µM reduced the expression of COX-2 and suppressed activation of protein kinase MAPK, including p38, ERK1/2, and JNK in THP-1 cells. The activation and nuclear translocation of NF-κB were also reduced after the treatment with procyanidin B2 as a consequence of the inhibition of the nuclear concentration of p65/p50 and prevention of LPS-induced degradation of IκBα [199]. Therefore, the inhibition of COX-2 might be due to the inhibition of phosphorylation of MAPK proteins as well as the prevention of DNA binding of NF-κB through the stabilization of IκB proteins [199]. In LPS-treated HUVEC, pretreatment with procyanidin B2 reversed LPS-mediated alterations to p-p65 NF-κB, p-IκBα, and p-IκBβ protein expression levels [200]. Similar observations were made in the case of procyanidin A1 in the RAW264.7 cell line [198]. Furthermore, in septic acute kidney injury in mice, the translocation of Nrf2 from the cytosol to the nucleus was observed in kidney tissue of animals treated with procyanidin B2 at doses of 50, 100, and 200 mg/kg. Therefore, it was concluded that procyanidin B2 alleviated ROS accumulation and reduced mitochondrial damage along with promoting mitochondrial biogenesis and improving mitochondrial dynamics [201].

**Table 2:** Revision of studies on the molecular role of certain phenolic compounds in vitro and in vivo; ↓ inhibition; ↑ activation.

Compounds	Model	Mode of Action	Reference
Lignans			
Nordihydroguaiaretic acid	human SH-SY5Y neuroblastoma cells	↓ ERK1/2	[173]
	I/R * model in rats	↑ Nrf2	[172]
Tannins			
Gallotannins and ellagitannins			
Castalagin	osteoclasts differentiated from bone marrow-derived macrophages (BMM)	↓ NF-κB, MAPK	[188]
Epicatechin gallate	cisplatin-induced nephrotoxicity in rats	↓ MAPK, IL-6, TNF-*α*	[194]
	cerebral edema in mice	↓ NF-κB, TNF-*α*, IL-1*β*	[190]
	atherosclerosis model in mice	↑ Nrf2, ↓ NF-κB, IL-6, TNF-*α*	[193]
Epigallocatechin gallate	RANKL-induced osteoclast differentiation in macrophage cell line (RAW 264.7)	↓ NF-κB, MAPK	[189]
	CoCl_2_-induced murine microglial cell line (BV2)	↑ Nrf2 ↓ NF-κB, IL-6, COX-2	[191]
	acute lung injury model in mice	↓ NF-κB, IL-6, TNF-α, IL-1*β*	[195]
Pentagalloylglucose	UVB-induced human dermal fibroblasts	↓ NF-κB, MAPK	[197]
	AGE **-induced mouse mesangial cells (MES)	↑ Nrf2, ↓ TNF-α, IL-1*β*	[196]
Procyanidins			
Procyanidin A1	LPS-induced murine macrophage cell line (RAW 264.7)	↑ Nrf2 ↓ NF-κB, MAPK, IL-6, TNF-α, NO	[198]
	LPS-induced human umbilical vein endothelial cells (HUVECs)	↓ NF-κB, IL-6, TNF-α, IL-1*β*	[200]
Procyanidin B2	LPS-induced human monocyte cell line (THP-1)	↓ NF-κB, MAPK, COX-2	[199]
	acute kidney injury model in mice	↑ Nrf2	[201]
Phenolic glycosides			
Salicin	LPS-induced murine macrophages RAW 264.7	↓ NF-κB, MAPK, IL-6, TNF-*α*, IL-1*β*	[202]
	rheumatoid arthritis fibroblast-like synoviocytes (RA-FLSs)	↑ Nrf2, ↓ NF-κB, IL-1*β*, TNF-*α*, MMP-1, MMP-3	[203]
Phenolic acids and depsides			
Caffeic acid	LPS endothelial cell line (YPEN-1)	↓ NF-κB, COX-2	[204]
	LPS-induced primary bovine mammary epithelial cells	↓ NF-κB, MAPK, IL-1*β*, IL-6, TNF-*α*, IL-8	[205]
	*t*-BHP ***-induced HepG2 cells	↑ Nrf2	[206]
Chlorogenic acid	porcine jejunal epithelial cell line (IPEC-J2)	↓ NF-κB, TNF-*α*	[207]
	endometriosis model in mice	↑ Nrf2, ↓ NF-κB, IL-6, TNF-*α*, IL-1*β*	[208]
	DSS ****-induced ulcerative colitis in mice	↓ MAPK, IL-6, TNF-*α*, IL-1*β*	[209]
Cichoric acid	neuroinflammation model in mice	↓ NF-κB, MAPK, COX-2, TNF-*α*, IL-1*β*	[210]
	LPS-induced murine microglial cell line (BV2) and mice brain	↑ Nrf2, ↓ NF-κB, MAPK	[211]
*p*-Coumaric acid	doxorubicin-treated cardiomyoblast cell line (H9C2)	↑ Nrf2	[212]
	human lens epithelial cell line (HLE SRA 01/04)	↓ MAPK	[213]
Curcumin	LPS-induced murine microglial cell line (BV2)	↓ NF-κB, IL-6, IL-1β	[214]
	H_2_O_2_-induced murine macrophage cell line (RAW 264.7)	↓ Nrf2	[215]
	influenza A virus infection in lung cancer cell line (A549)	↑ Nrf2,↓ NF-κB, MAPK, MMP-2, MMP-9	[216]
Ellagic acid	CCl_4_-induced pancreas damage in rats	↑ Nrf2, ↓ NF-κB, TNF-*α*	[217]
	rotenone-induced neurotoxicity in mice, neuronal cell lines (MN9D, BV2, C6)	↑ Nrf2	[218]
	TNF-*α*/IFN-*γ*-induced keratinocyte cell line (HaCaT)	↓ MAPK, TNF-*α*, IL-6	[219]
Ferulic acid	LPS-induced bovine endometrial epithelial cell line (BEEC)	↓ NF-κB, MAPK, IL-6, TNF-α, IL-1*β*, IL-8	[220]
	LPS-induced primary bovine mammary epithelial cells	↑ Nrf2, ↓ NF-κB	[221]
	acute lung injury model in mice	↓ NF-κB, IL-6, TNF-*α*, IL-1*β*	[222]
Gallic acid	elastase-induced emphysema in rats	↑ Nrf2, ↓ NF-кB	[223]
	ethanol-induced gastric ulcer in rats	↑ Nrf2 ↓ IL-1*β*, IL-6	[224]
	diabetic nephropathy model in rats	↓MAPK, IL-1*β*, IL-6, TNF-*α*	[225]
Rosmarinic acid	human hepatoma cell line (HepG2)	↓ NF-κB, MMP-2, MMP-9	[226]
	acute liver injury model in mice	↑ Nrf2, ↓ NF-κB, MAPK	[227]
	spinal cord injury model in rats	↑ Nrf2, ↓ NF-κB, IL-6, TNF-*α*, IL-1*β*	[228]
Phenylpropanoids			
Echinacoside	IL-1*β* -induced rat chondrocytes	↑ Nrf2	[229]
Verbascoside	LPS-induced murine microglial cell line (BV2)	↓ NF-κB, IL-6, IL-1*β*	[230]
Stilbenoids			
Resveratrol	porcine jejunal epithelial cell line (IPEC-J2)	↑ Nrf2	[231]
	fibrosis model in mice	↓ NF-κB	[232]
	IL-1*β*-stimulated rat synovial cells (RSC-364)	↓ MAPK	[233]

* I/R—ischemia–reperfusion; ** AGE—advanced glycation end products; *** *t*-BHP—*tert*-butyl hydroperoxide; **** DSS—dextran sulfate sodium.

## 6. Phenolic Glycosides

Salicin (2-(hydroxymethyl)phenyl-*β*-d-glucoside) is well-known as a precursor of a common anti-inflammatory drug: aspirin. The best-known source of salicin is the bark of *Salix* spp. as well as the stems and roots of *Alangium chinense* (Lour.) Harms (Cornaceae) [202,203]. D(−)-Salicin in concentrations of 35, 70, and 140 µM inhibited LPS-induced activation of p65, p38/MAPK, JNK, and ERK in a concentration-dependent manner in RAW264.7 cells. In particular, p38/MAPK led to the activation of NF-κB. This mode of action was indicated as responsible for the inhibition of pro-inflammatory cytokines such as TNF-*α*, IL-1*β*, and IL-6 both in RAW264.7 cells and in BALF of LPS-induced ALI mice [202]. Apart from inhibition of phosphorylated p65, salicin in the concentration range of 1.2–120 µM promoted nuclear translocation of Nrf2 and HO-1 expression in IL-1*β*-induced rheumatoid arthritis fibroblast-like synoviocytes (RA-FLSs). In addition, it regulated oxidative stress markers in collagen-induced rat joint tissue [203]. Taking into consideration that osteoarthritis might be related to AGE, it was shown that salicin inhibited AGE formation in human SW1353 chondrosarcoma cells as well as AGE-induced expression and secretion of TNF-*α*, IL-1*β*, MCP-1, and HMGB-1 through the inhibition of the AGE-induced activation of NF-κB. The nuclear p65 was reduced by salicin at concentrations of 50 and 100 µM [234]. The role of salicin in osteoporosis was also studied. It is worth noting that NF-κB is an important pathway during osteoclastogenesis. RANKL-induced differentiation of osteoclasts and the activity of NFATc1 in vitro was suppressed by salicin [235]. Similar molecular effects were observed in retinal endothelial cells (RECs) and human umbilical vein endothelial cell-originated cells (ECV304) [236,237].

## 7. Phenolic Acids and Depsides

**Figure 4:**
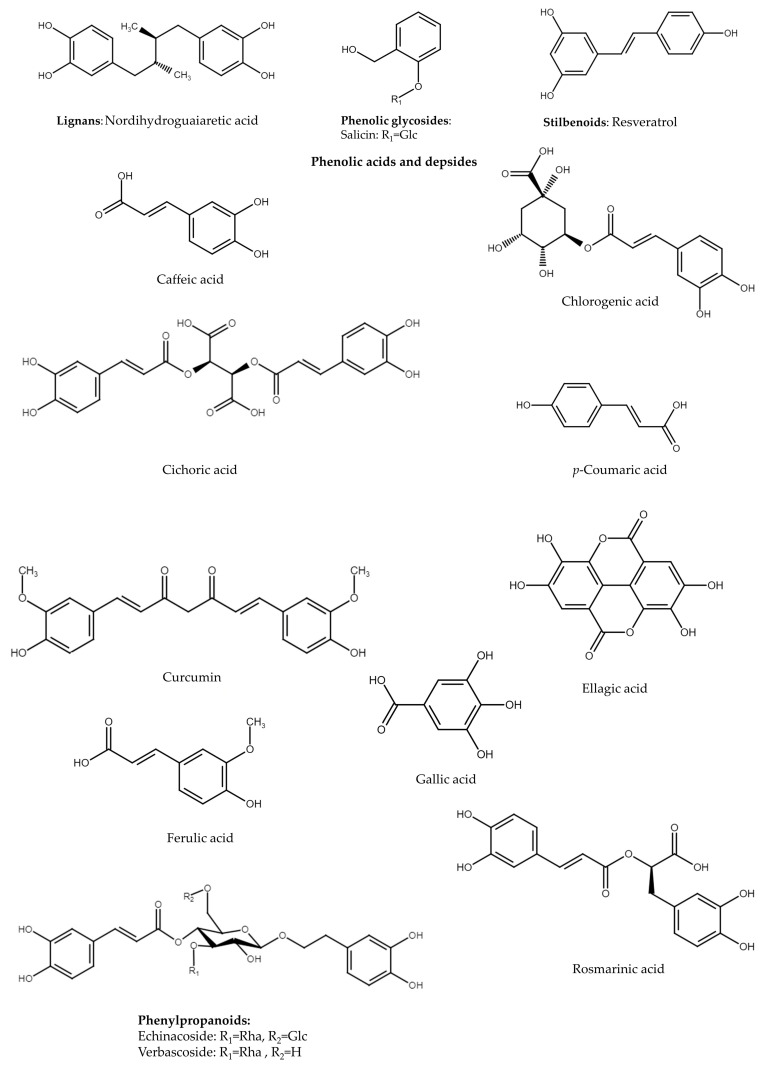
The structures of phenolic compounds listed in Table 2. Glc—glucose; Rha—rhamnose.

The group of phenolic compounds, known as aromatic secondary metabolites, is particularly widespread in the plant kingdom. These compounds contain both hydroxyl and carboxyl groups (Figure 4). Phenolic acids include hydroxyl derivatives of benzoic (gallic acid, protocatechuic acid, salicylic acid) and cinnamic acids (caffeic acid, cinnamic acid, *p*-coumaric acid, ferulic acid). They occur mainly in their bound forms of glycosides as the aforementioned phenolic glycosides and in the form of esters as depsides and depsidones such as chlorogenic acid, curcumin, ellagic acid, and rosmarinic acid. The environmental factors, including climatic conditions, cultivation, fertilization, and time of harvest have a great impact on their presence in plant materials. It is worth noting that plants from Lamiaceae, fruits, and vegetables contain significant amounts of phenolic acids. In addition, other important factors determining phenolic acid concentration are storage conditions of the plant materials and the methods of preparation. The main sources of phenolic acids are herbal infusions, including black and green tea, and coffee [238]. The role of isolated/pure phenolic acids in inflammation-related molecular pathways was confirmed in atopic dermatitis, lung injuries, neurodegenerative diseases, and chronic liver and pancreas disorders, as well as inflammatory diseases within the epithelium or endothelium.

Ellagic acid in the concentration range of 250 to 1000 µM suppressed MAPK pathways in a keratinocyte cell line (HaCaT) stimulated with TNF-*α*/IFN-*γ*. In addition, the compound inhibited the phosphorylation of JAK or STAT proteins, which are involved in the inflammatory activity of cytokines in atopic dermatitis, and pSTAT(Y) nuclear translocation. The improvement of skin lesions in atopic dermatitis induced with *Dermatophagoides farinae* extract (DfE) was observed in NC/Nga mice treated with ellagic acid (40 mg/kg) for 28 days [219]. The effect of phenolic compounds on respiratory tract diseases was assessed in acute lung injury models. Both ferulic acid and curcumin influenced the inactivation of TLR4/NF-κB and MAPK signaling pathways in lung tissues injured by LPS or influenza A virus (IAV) [216,222]. Ferulic acid (25–100 mg/kg) reduced the cytokine level in the bronchoalveolar lavage fluid and TLR4, p-p65, and p-IκBα in the lung tissues of mice [222]. In addition, curcumin induced Nrf2 and suppressed the phosphorylation of p38, ERK, and JNK in IAV-stimulated A549 cells. The anti-inflammatory effect was also observed in vivo. Curcumin decreased the levels of pulmonary cytokines (TNF-*α*, IL-1*β*, IL-6, IL8) and the levels of pulmonary metalloproteinases (MMP-2, MMP-9). Additionally, it improved IAV-induced pulmonary histopathological changes [216]. The significance of curcumin in viral infections and related inflammation was recently reviewed by Šudomová & Hassan (2021). Curcumin has been shown to inhibit herpes simplex virus (HSV-1 and HSV-2) adsorption and replication. It was reported that curcumin inhibited the replication of HSV-2 through the NF-κB pathway. Additionally, its antiviral properties were established in other human and animal herpesviruses, including human cytomegalovirus, Kaposi’s sarcoma-associated herpesvirus, Epstein–Barr virus, bovine herpesvirus 1, and pseudorabies virus, with various assays [239].

Next, the anti-inflammatory activity of phenolic acids and depsides in inflammatory perturbations of the liver or pancreas was proved. For example, acute liver injury in mice was induced with LPS (30 μg/kg) and D-galactosamine (600 mg/kg). In this model, rosmarinic acid at doses of 25, 50, and 100 mg/kg induced HO-1 and NQO1 expression, whereas the expressions of p-p65, p-JNK1/2, p-ERK1/2, and p-p38 were decreased [227]. Additionally, rosmarinic acid at concentrations of 100, 200, and 400 µM inhibited the expression of MMP-2 and MMP-9 in the hepatoma cell line HepG2 [226]. In the same cell line model, caffeic acid particularly increased the nuclear translocation of Nrf2 and HO-1 expression. The influence of this compound on MAPK expression was visualized with western blot [206]. Rosmarinic acid also influenced HepG2 cell apoptosis through the increased expression of cleaved caspase-3 and Bax and the reduced expression of Bcl-2 [226]. Similar observations were provided for ellagic acid, which was administered to rats. When the compound (10 mg/kg) was injected intraperitoneally, it slightly regulated the ADS enzymes and NF-κB, Bcl2, and Nrf2 signaling pathways in pancreas tissue [217].

A wide range of reports on phenolic acids in epithelial inflammation was provided. The epithelium is usually the first tissue having contact with microorganisms or plant-derived products, as in the gastrointestinal tract. Chlorogenic acid is considered the most abundant phenolic acid in dietary products. It was identified as the agent that decreases intestinal permeability and ameliorates intestinal injury in rats and pigs. The background mechanism of this activity was further established in a porcine jejunal epithelial cell line (IPEC-J2) stimulated with diquat or TNF-*α*. In general, chlorogenic acid (100 µM) enhanced Nrf2 and HO-1 levels on the one hand, and reduced phosphorylation in IκBα/NF-κB pathway on the other hand. Indeed, this effect was visualized in a relevant manner [207]. Next, in primary bovine mammary epithelial cells (bMEC), caffeic acid inhibited the LPS-induced phosphorylation levels of JNK, p38, and c-Jun and significantly blocked the LPS-activated phosphorylation of ERK1/2 [205]. In the same model, ferulic acid slightly influenced NF-κB and Nrf2 signaling pathways [221]. Furthermore, ferulic acid (40, 80, and 120 µM) particularly suppressed the phosphorylation of MAPK and p65 proteins in comparison with the LPS-stimulated control of bovine endometrial epithelial cells [220]. In addition, both caffeic and ferulic acids reduced the rate of bMEC damage induced by LPS by protecting against apoptosis and inhibiting mitochondrial dysfunction and ROS generation [205,221]. It is worth noting that bMECs are responsible for milk formation during lactation. However, they also contribute to the early response of the *E. coli*-infected mammary gland, as well as promote neutrophil recruitment and participate in tissue inflammation during *E. coli* mastitis [205]. Similarly, infection of *E. coli* is the main reason for bovine endometritis [220]. Therefore, the significance of the correlation between the presence of phenolic compounds in a diet and medicinal plants and inflammation is considered not only in human but also veterinary therapy. Apart from microbial pathogens, oxidative stress is the second main reason for tissue dysfunction. The antioxidant significance of *p*-coumaric acid was established in human lens epithelial cells (HLE) stimulated with H_2_O_2_ (275 µM) in vitro. The compound in concentrations of 3, 10, and 30 µM decreased the phosphorylation of p38, ERK, and JNK. The enzymes of the ADS were activated after the treatment with *p*-coumaric acid in HLE as well as in myoblast cells derived from the rat myocardium (H9c2) [213]. In this last model, *p*-coumaric acid significantly induced Nrf2 expression [212].

To summarize the effect of phenolic acids in the model related to cardiovascular disorders, we focused on the reports on endothelial cells or monocytes/macrophages. Firstly, caffeic acid at concentrations of 10 and 50 µM reduced the expression of COX-2 and phosphorylation of p65, IKKα/β, and ERK1/2 in an endothelial cell line (YPEN-1) stimulated with LPS [204]. Secondly, the activity of curcumin was in opposition to previous reports on the effect of phenolic compounds on the Nrf2 signaling pathway. Curcumin at concentrations of 5, 10, and 20 µM significantly suppressed the mRNA levels of Nrf2 and Keap1 in comparison with the H_2_O_2_-treated RAW264.7. The authors concluded that this effect was related to posttranscriptional control [215].

Considering the influence of phenolic compounds in neuroinflammation, a model of BV2, a microglial cell line, was often used. The effects of curcumin (1–10 µM) and cichoric acid (80 µM), also named chicoric acid, on p65 NF-κB, IκBα, Nrf2, HO-1, NQO-1, MAPK, COX-2, and/or iNOS in BV cells were studied [211,214]. When C57BL/6J mice were treated with 0.05% cichoric acid, the compound prevented LPS-induced increases in *β*-amyloid accumulation [210]. Cichoric acid prevented LPS-induced NF-κB and MAPK activation in mouse brains [210,211]. The neuroprotective properties were reported for rosmarinic and ellagic acids. Rosmarinic acid inhibited apoptosis in the spinal cord of injured (SCI) rats. The screening of molecular pathways, including Nrf2, p-IκBα, TLR4, and MyD88, was also provided in this study. In general, the nucleus level of Nrf2 was increased, whereas the nucleus level of NF-κB was decreased in the groups treated with rosmarinic acid at doses of 10, 20, and 50 mg/kg [228]. Ellagic acid at doses of 20 and 100 mg/kg increased protein expression of nuclear Nrf2 as well as HO-1 and NQO1 in rotenone-induced mice brains. The effect of ellagic acid on the activation of nuclear Nrf2 and the decrease in Nrf2 in the cytosol was confirmed in vitro in different neuron–microglial and neuron–astroglial cells such as MN9D, BV2, and C6. Therefore, the compound might be a beneficial agent for neurodegenerative disease treatment [218].

The molecular pathways considered in this review were also target points for gallic acid. Its effectiveness was established in vivo. Firstly, gallic acid was tested as a potential agent for controlling inflammation and preventing the progression of chronic obstructive pulmonary disease. Emphysema is associated with destructive changes in the alveolar walls and chronic inflammation due to the breakdown of the extracellular matrix parenchyma. The significantly increased expression of Nrf2 and HO-1 was observed in gallic acid (30 mg/kg)-administered rats compared with the porcine pancreatic elastase-treated group (control). On the other hand, relative NF-κB mRNA expression was decreased in lung tissues [223]. Secondly, the compound exerted a protective effect on gastric mucosa, where Nrf2 and HO-1 expressions were increased, in ethanol-induced gastric ulcers in rats [224]. Thirdly, gallic acid showed protective activity in diabetic nephropathy in rats. While the animals were stimulated with HFD and streptozotocin, gallic acid at doses of 25 and 50 mg/kg suppressed the phosphorylation of p38 and p65 [225]. It is worth noting that gallic acid, in addition to its occurrence in plant materials, may be an important metabolite derived from the digestion of macromolecular compounds such as gallotannins [72].

Recently, ellagic acid was established as a promising agent in collagen-induced arthritis in rats. In this model, the effectiveness of ellagic acid on inflammatory mediators (NF-κB, iNOS, TNF-*α*, IL-1*β*, IL-6, and IL-10) and oxidative-stress-related parameters (MPO, NO, LPO, catalase, SOD, GSH) was studied [240].

## 8. Phenylpropanoids

The shikimate pathway provides phenylalanine and the entry point leading to the biosynthesis of phenylpropanoids along with coumarins, stilbenoids, flavonoids, and lignans [13,241]. The most common phenylpropanoids seem to be echinacoside and verbascoside (syn. acteoside, kusaginin, or orobanchin). They were isolated from *Echinacea angustifolia* DC. (Asteraceae) in 1950 and *Verbascum sinuatum* L. (Scrophulariaceae) in 1963, but their structures were determined in 1983 and 1968, respectively [242].

Echinacoside in the concentration range of 2.5 to 10 mg/mL significantly decreased the expression of TREM2 (triggering receptor expressed on myeloid cells 2) in human renal tubular epithelial cells (HK-2) and reduced the translocation of NF-κB to the nucleus in overexpressed-TREM2 HK-2 cells. In addition, echinacoside disrupted the function of hepatitis B virus X in HK-2 cells. It was concluded that hepatitis B virus X may be affected by echinacoside as its suppressor in HK-2 cells [243]. In male C57BL/6 mice, echinacoside at a dose of 30 mg/kg/day diminished the MPTP (1-methyl-4-phenyl-1,2,3,6-tetrahydropyridine)-induced expression of phospho-p38 MAPK and phospho-NF-κB p52 compared to the MPTP group after 14 days of treatment. It must be underlined that this effect was comparable with minocycline, which is a known inhibitor of neuroinflammation. It is worth noting that the phosphorylation of p38 MAPK promotes the death of dopaminergic neurons. In addition, the administration of echinacoside in a dose of 50 mg/kg/day for 3 months caused the activation of the PI3K/AKT/Nrf2/PPAR*γ* pathway, a decrease in ROS formation, as well as an upregulation of SOD1/2 in APP/PS1 mice. Consequently, the alleviation of memory impairment was observed in animals [244]. Echinacoside also inhibited the IL-6/JAK2/STAT3 pathway and reduced the phosphorylation of STAT3 in BV2 cells. Taking into consideration that cytokines such as TNF-*α* might be activators of NF-κB in neurons, it is crucial to reduce their concentration in the innate, nonspecific, immune response. Indeed, echinacoside decreased the relative mRNA level expression of IL-1*β*, TNF-*α*, IL-8, and IL-6 in MPTP-treated mice in vivo as well as in IL-1*β*-induced rat chondrocytes in vitro [229,245]. Taking together, the inhibition of NF-κB, MAPK, as well as the activation of Nrf2 pathways, support the statement that echinacoside participates in the regulation of neuroinflammation [246]. On the other hand, pyroptosis is a programmed cell death determined by an inflammatory pathway derived from impaired mitochondria and the activated inflammasome. It is believed that pyroptosis is associated with inflammation responses in tumor cells. Echinacoside (0–100 µM) inhibited the signaling of one of the MAPK pathways, Raf/MEK/ERK, in non-small cell lung cancer (NSCLC), the activation of which is associated with tumorigenic diseases. The inhibition of this pathway was indicated as important for the induction of pyroptosis [247]. Apoptosis and chronic inflammation in bladder tissue are characteristics of interstitial cystitis. In this case, echinacoside decreased the phosphorylation levels of IκB and p65 NF-κB and upregulated the expression of PPAR*γ*, which are related to apoptosis and inflammation in cyclophosphamide-induced mouse cystitis [248]. For chronic inflammation, echinacoside at a dose of 30 mg/kg/day influenced chondrocyte injury in rats by the Nrf2/HO-1 signaling pathway. In this manner, the progression of osteoarthritis was alleviated [229].

The effect of verbascoside on cell survival, antioxidant enzyme activity, as well as NF-κB mediated inflammatory mediator production was established in a few models of human lung carcinoma A549 cell line, RAW-Blue cells, LPS-induced BV2 cells, and A*β*1-42-stimulated mouse-derived neuroblastoma cells (N2a) [230,249,250]. The effective concentration for inhibition of NF-κB/AP-1 activity in RAW-Blue cells was 10.08 µM (55.55% inhibition) [250]. Verbascoside changed the expression of sorbin and SH3 domain-containing protein 2 (SORBS2) and plexin-B2 (PLXNB2), which was linked with cytokines and p65 of NF-κB signaling in the brains of APP/PS1 mice, LPS-stimulated BV2 cells, and A*β*1-42-induced N2a cells. Verbascoside reversed the overexpression of TGF-*β*, IL-1*β*, and MCP-1, which is an inflammatory-activation chemokine involved in recruiting monocytes and promoting inflammation. The levels of IL-6 and iNOS were also reduced, whereas verbascoside boosted the production of anti-inflammatory factors, including IL-4 and IL-10. Moreover, verbascoside effectively inhibited the phosphorylated levels of IKKα, IKKβ, and p65 NF-κB in the brains of APP/PS1 mice [230]. Apart from neuroinflammation, this molecular pathway was indicated as a mode of action in osteoarthritis in rats [251].

## 9. Stilbenoids

Resveratrol (*trans*-3,5,4′-trihydroxystilbene) is a polyphenol often occurring in grapes—particularly, the skin of red grapes—and berries, peanuts, mulberry, and medicinal plants of the *Polygonum* species, particularly in the roots of *Polygonum cuspidatum* Siebold & Zucc. (*Reynoutria japonica* Houtt., Polygonaceae) [233,252]. Resveratrol strongly inhibited the formation of arachidonic acid derivatives such as leukotrienes B4 and C4 as well as thromboxane B2 via inhibition of 5-LOX and COX. In addition, it influenced arachidonic-acid-induced platelet aggregation [252]. The background for the biochemical mode of anti-inflammatory action of resveratrol depends at least partially on the inhibition of NF-κB and MAPK signaling in addition to the activation of PI3K/Akt-mediated Nrf2 signaling, which was proved both in vitro and in vivo [231,232,233]. Resveratrol influenced SOD and MDA levels as well as attenuated inflammation in a bovine type-II collagen (BIIC)-induced Sprague−Dawley rat arthritis model [233]. In porcine intestinal epithelial cells (IPEC-J2), resveratrol increased relative mRNA expression of tight junctions such as claudin-1, occludin, and ZO-1, strengthening the intestinal barrier [231].

## 10. Other Compounds Related to Chronic Inflammation

The last compounds we discuss are the coumarins. The biosynthesis of coumarins takes place in the shikimate pathway from phenylalanine, which is transformed into *trans*-cinnamic acid. The central metabolite of the shikimate pathway is 4′-coumaroyl-S-CoA. In addition to other phenolic compounds, it gives rise to coumarins due to subsequent reactions such as 6′-hydroxylation, the *trans* > *cis* isomerization of the exocyclic double bond, and the final lactonization/cyclization. Next, the 3,4-epoxide may be transformed into 3-hydroxycoumarin and then into *o*-hydroxyphenyl lactic acid and its derivatives. The possible metabolites of coumarin are the 3,4-dihydrocoumarin and hydroxylated coumarins. In this last case, hydroxylation is most often at position 7 and rarely at positions 4, 5, 6, and 8 [253]. Since *Hippocastani cortex* from *Aesculus hippocastanum* L. (Sapindaceae) is a traditional herbal medicinal product for the relief of symptoms of discomfort and heaviness of legs related to minor venous circulatory disturbances, as well as for symptomatic relief of itching and burning associated with hemorrhoids, the role of esculin as a representative coumarin in the inflammation is worth noting. The content of glycosidic coumarins such as esculin, fraxin, and scopolin in horse chestnut bark can reach 7%. The corresponding aglycones include esculetin, fraxetin, and scopoletin [254].

Most of the protective effects of esculin were assigned to the inhibition of NF-κB activation in a HepG2 cell line [255], dextran sulfate sodium (DSS)-induced mice and RAW264.7 macrophages [256], LPS-induced acute injury in mice [257], and in RANKL-induced osteoclastogenesis of RAW264.7 macrophages [258].

The anti-inflammatory effect of esculetin was related to its inhibition on the activation of NF-κB and MAPK signaling pathways both in vitro and in vivo in LPS-induced RAW264.7 cells, DSS-induced colitis in mice [259], and LPS-induced acute lung injury in mice [260] and alleviated the progression of lupus nephritis MRL/lpr mice via activation of the Nrf2 signaling pathway [261]. In addition, it attenuated the phosphorylation of ERK1/2 and NF-κB expression mediated by LPS in human RPE cells concerning age-related macular degeneration [262].

The plant extracts from the leaves of *Eucalyptus globulus* Labill. (Myrtaceae) are traditionally used for the treatment of symptoms of respiratory infections, such as cold, flu, and sinusitis. Tereticornate A, a triterpenoid compound isolated from the ethanolic extract from leaves of *E. globulus*, showed a significant anti-HSV-1 potential (IC_50_ = 0.96 μg/mL). It significantly inhibited the activation of NF-κB as well as attenuated the LPS-stimulated secretion of IL-1*β* in THP-1 macrophages. This might provide support for its effectiveness and role in the viral infections of respiratory tracts [263].

Finally, xanthones (xanthene-9-one), which include *α*-mangostin and *γ*-mangostin (*Garcinia mangostana* L., Clusiacaceae), have been considered to play a role in obesity-associated inflammation [264,265]. Both *α*-mangostin and *γ*-mangostin attenuated phosphorylation of Jun, ERK, and p38. Mangostins also attenuated LPS-induced IκBα degradation and NF-κB activation in human adipocytes and U937 monocytes [264,265]. The anti-inflammatory properties of xanthones as well as the possibilities of their application in skin inflammatory diseases such as atopic dermatitis and psoriasis were the subject of previous detailed reviews [266,267].

## 11. Plant Materials and Network Pharmacology

We believe that the referred data concerning the molecular targets for phenolic compounds are justified. Indeed, isolated compounds regulate inflammation in NF-κB, Nrf2, and MAPK signaling pathways. However, these pathways are related to different chronic disorders such as osteoarthritis, neuroinflammation, cardiovascular, or gastrointestinal diseases, depending on the model developed in the study. For this reason, the effect of phenolic compounds may be systemic and not specific to the disease. The concept of selective ligand design acting on specific targets is the essential paradigm of drug discovery. However, many effective drugs modulate multiple proteins rather than single targets [268]. The growing amount of evidence in this field provides the background for different advanced databases (Table 3). Over the past few years, the foundation of network pharmacology in drug discovery has been developed. This concept of network pharmacology allowed for a more powerful analysis of complex plant-derived drugs like those used in traditional Chinese medicine [269]. Advances in biological systems revealed that selective compounds, compared with multitarget drugs, may exhibit lower than desired clinical efficacy. Therefore, the paradigm shifted from a “one-target, one-drug” mode to a “network-target, multiple-component-therapeutics” mode in network pharmacology [268].

The significance of phenolic compounds mentioned in this review for this concept is particularly notable. Complex networks reflecting the relationship between active compounds and targets are constructed and the protein–protein interaction network is analyzed using software and databases. Based on these network analyses, phenolic compounds such as quercetin can be commonly indicated for many herbal formulations. Network pharmacology, molecular docking, and experimental validation in vivo were used to provide evidence for ingredients of Chinese formulations (Wumei Pill) such as Fructus Mume (FM, *Prunus mume* Sieb.et Zucc., Rosaceae) and Rhizoma Coptidis (RC, *Coptis chinensis* Franch., Ranunculaceae) in the treatment of ulcerative colitis. For these two plant materials, six components of FM, including *β*-sitosterol, stigmasterol, kaempferol, quercetin, and 13 components of RC, including quercetin, berberine, berberrubine, epiberberine, palmatine, and coptisine, were retrieved from the systematic pharmacology analysis platform (TCMSP). In the molecular docking, it was established that quercetin and MAPK1 proteins had quite a small docking energy, lower than kaempferol. However, mass spectral data of five ingredients in FM-RC—citrate, citric acid, jatrorrhizine, palmatine, and berberine—were particularly pronounced during UPLC-QTOF-MS analysis. The pharmacological experiments showed that the FM-RC formulation restored intestinal functions after DSS-induced damage in rats [270].

Similarly, flavonoids, triterpenoids, organic acids, amino acids, tannins, phenolic acids, and coumarins were found in the fruit of *Rosa roxburghii* Tratt. (Rosaceae) using UPLC-Q-Exactive Orbitrap/MS after a preliminary screening of databases. The authors emphasized that by comparing the bioinformatic and spectral tools, 30 compounds were confirmed in both methods, whereas 49 and 24 different compounds were found in databases and UPLC-Q-Exactive Orbitrap/MS, respectively. In addition, four compounds were unknown. In this study, compound–network construction was performed to explore the mechanisms of *Rosa roxburghii* fruit in diabetes mellitus treatment. The core targets, including AKT1, TNF, VEGFA, MAPK1, and MAPK3, were established for this plant material in the treatment of diabetes mellitus based on network analysis [271]. Furthermore, the ingredients–targets–rheumatoid-arthritis network showed that quercetin, kaempferol, ferulic acid, and *p*-coumaric acid can be found in *Hedyotis difusa* Willd. (Rubiaceae) and that they are crucial phytochemicals for the treatment of rheumatoid arthritis [272]. Based on network construction, quercetin, kaempferol, isorhamnetin, luteolin, and formononetin were also indicated as active components of the Buzhong Yiqi Decoction (BZYQD) that is used in cases of osteoporotic fracture. The treatment of osteoporotic fracture with BZYQD, which is composed of ten medicinal plant materials, was mainly related to the TNF signaling pathway, ROS, NF-κB, and MAPK signaling pathway. The aqueous extract of BZYQD promoted fracture repair in rats by regulation of NF-κB and the MAPK signaling pathway in femoral tissue [273].

Therefore, even though the analysis of network pharmacology indicates targets for phytochemicals, detailed experimental studies are still required. There is no doubt that the analysis of network databases and the use of bioinformatics tools, including molecular docking, should be the first step in drug design processes, followed by experimental procedures. Nevertheless, with relation to plant materials, even though the phytochemicals are selected based on the network analysis, their detailed analysis in plant materials using traditional analytical tools must be included to assure the quality of tested plant materials, particularly in the complex herbal formulations. It is worth noting that phenolic compounds are widely distributed in plant materials in general. Network pharmacology and bioinformatics tools can be considered important sources of preliminary data. However, the results of these analyses must be taken carefully into consideration, since some compounds or groups of compounds, in particular flavonoids, were often included in the component–target results even though different plant materials were studied. The preparation of plant materials using specific solvents determines the presence or absence of phytochemicals retrieved from databases. The qualitative and quantitative phytochemical analysis of plant preparations along with pharmacological experiments seems to be the only way to confirm the results of network analysis. Both these pathways should be strictly correlated. While the analytical tools are still necessary to discover specific compounds of complex herbal formulations, pharmacological effects can be confirmed by experimental validation in vivo.

## 12. Conclusions

A wide range of studies show that many plant-derived secondary metabolites could down-regulate the expression and production of inflammatory mediators and their receptors in chronic diseases. This prevention of chronic inflammation is due to the activation of the expression of transcription factors, mainly NF-κB and Nrf2, which participate in the secretion of inflammatory mediators or antioxidant defense. Taking into consideration the intake of phenolic compounds from dietary sources, this could be the simplest and safest way to prevent or support the therapy of chronic diseases as well as modulate activities in different inflammatory pathways. It is worth noting that many molecular pathways are engaged in the pathogenesis of chronic inflammatory diseases, including neuroinflammation-related neurodegenerative diseases, osteoarthritis, or atherosclerosis. Therefore, long-term intake of polyphenolic compounds may support therapeutical procedures and prevent the development of disease progression. The consumption of polyphenol-rich foods may contribute to achieving optimal health benefits as well as play an important role in reducing the risk or delaying the development of cardiovascular disease, cancer, and other age-related diseases.

Finally, bioinformatic tools such as network pharmacology are increasingly used for the prediction of the pharmacology effect of plant materials and their active phytochemicals. However, in silico studies should be carefully treated as the first step of pharmacological hypotheses verification. Due to the diverse molecular targets for phenolic compounds in multi-component plant materials, their pharmacological effects may not be obvious. Many molecular pathways, such as those revised in this paper, are engaged in different pathological conditions, and they are considered as background for many chronic diseases. In addition, the synergistic or additive interaction of phytochemicals in plant extracts may potentiate the pharmacological effect. Experimental validation of the bioinformatic and network analyses is still required. Due to the complex nature of plant-derived products, the standardization of the multi-component preparations is necessary to assure the quality of natural products, particularly medicinal plants.

## Figures and Tables

**Table 3:** Number of records in Scopus, PubMed, and Medline databases for selected phenolic compounds and keywords such as NF-κB, Nrf2, and MAPK.

Compounds	Scopus			PubMed			Medline		
	NF-κB	Nrf2	MAPK	NF-κB	Nrf2	MAPK	NF-κB	Nrf2	MAPK
Apigenin	354	213	290	286	88	138	346	113	270
Diosmetin	33	24	17	14	14	7	13	13	9
Diosmin	26	12	16	19	8	5	149	46	123
Luteolin	375	292	371	305	127	175	350	157	292
Vitexin	44	35	30	28	15	10	23	12	14
Avicularin	8	1	3	10	1	2	8	1	2
Hyperoside	42	28	23	43	28	20	34	23	21
Isoquercitrin	37	27	31	24	17	16	18	14	17
Isorhamnetin	88	64	90	51	21	34	37	14	33
Kaempferol	360	213	399	272	72	173	190	54	173
Quercetin	972	877	885	827	390	417	572	320	435
Rutin	146	86	101	181	83	65	114	58	65
Spiraeoside	-	1	1	-	1	1	-	1	1
Tiliroside	5	6	9	5	4	4	5	4	4
Eriodictyol	28	46	34	18	17	14	15	17	13
Hesperidin	142	120	108	14	61	44	94	54	47
Naringenin	169	132	169	119	51	58	47	44	59
Taxifolin	60	51	44	40	27	14	31	22	17
Catechin	539	361	407	603	236	282	391	206	310
Epicatechin	169	142	142	610	238	292	79	47	64
Formononetin	75	51	99	43	19	34	34	18	35
Genistein	553	231	668	489	83	484	289	70	614
Isosalipurposide	3	3	-	1	1	-	1	1	1
Xanthohumol	53	61	32	52	38	10	33	34	9
Silibinin	131	77	79	115	33	41	99	36	60
Cyanidin	110	84	84	109	47	48	40	24	25
Delphinidin	49	25	51	43	8	27	29	7	29
Malvidin	25	19	24	23	10	10	10	5	8
Pelargonidin	24	18	28	11	7	10	8	5	8
Nordihydroguaiaretic acid	47	22	34	39	11	25	17	9	30
Castalagin	3	1	-	2	-	-	2	-	-
Epicatechin gallate	72	57	63	22	10	16	12	7	20
Epigallocatechin gallate	526	427	399	367	142	191	235	127	213
Pentagalloylglucose	2	2	4	12	5	9	8	4	9
Procyanidin A1	5	1	5	2	1	2	1	1	3
Procyanidin B2	36	27	18	28	16	7	18	15	6
Salicin	7	5	4	10	5	3	7	5	3
Caffeic acid	535	249	248	405	109	96	340	120	137
Chlorogenic acid	260	182	164	196	92	70	148	76	77
Cichoric acid	17	20	10	25	12	9	5	1	1
Coumaric acid	169	131	111	153	79	63	116	68	69
Curcumin	1797	1165	855	1691	499	453	1192	429	507
Ellagic acid	155	104	88	123	46	33	99	35	39
Ferulic acid	181	182	156	125	81	60	94	74	65
Gallic acid	342	223	242	344	114	106	197	78	88
Rosmarinic acid	118	104	77	101	47	38	75	44	39
Echinacoside	14	10	12	9	9	4	7	5	3
Verbascoside	23	3	4	45	12	12	18	1	5
Resveratrol	1108	1046	676	995	394	346	711	342	361

## Data Availability

Not applicable.
